# Automated EuroFlow approach for standardized in-depth dissection of human circulating B-cells and plasma cells

**DOI:** 10.3389/fimmu.2023.1268686

**Published:** 2023-10-17

**Authors:** Alejandro H. Delgado, Rafael Fluxa, Martin Perez-Andres, Annieck M. Diks, Jacqueline A. M. van Gaans-van den Brink, Alex-Mikael Barkoff, Elena Blanco, Alba Torres-Valle, Magdalena A. Berkowska, Georgiana Grigore, J .J .M. van Dongen, Alberto Orfao

**Affiliations:** ^1^ Cytognos SL, Salamanca, Spain; ^2^ Translational and Clinical Research Program, Centro de Investigación del Cáncer (CIC) and Instituto de Biología Molecular y Celular del Cancer (IBMCC), CSIC-University of Salamanca (USAL), Salamanca, Spain; ^3^ Department of Medicine, University of Salamanca (USAL) and Institute of Biomedical Research of Salamanca (IBSAL), Salamanca, Spain; ^4^ Biomedical Research Networking Centre Consortium of Oncology (CIBERONC), Instituto de Salud Carlos III, Madrid, Spain; ^5^ Department of Immunology (IMMU), Leiden University Medical Center (LUMC), Leiden, Netherlands; ^6^ Centre for Infectious Disease Control, National Institute for Public Health and the Environment (RIVM), Bilthoven, Netherlands; ^7^ Institute of Biomedicine, University of Turku (UTU), Turku, Finland

**Keywords:** multiparameter flow cytometry, automated gating and identification approach, EuroFlow, standardization, B-cell, plasma cell

## Abstract

**Background:**

Multiparameter flow cytometry (FC) immunophenotyping is a key tool for detailed identification and characterization of human blood leucocytes, including B-lymphocytes and plasma cells (PC). However, currently used conventional data analysis strategies require extensive expertise, are time consuming, and show limited reproducibility.

**Objective:**

Here, we designed, constructed and validated an automated database-guided gating and identification (AGI) approach for fast and standardized in-depth dissection of B-lymphocyte and PC populations in human blood.

**Methods:**

For this purpose, 213 FC standard (FCS) datafiles corresponding to umbilical cord and peripheral blood samples from healthy and patient volunteers, stained with the 14-color 18-antibody EuroFlow BIgH-IMM panel, were used.

**Results:**

The BIgH-IMM antibody panel allowed identification of 117 different B-lymphocyte and PC subsets. Samples from 36 healthy donors were stained and 14 of the datafiles that fulfilled strict inclusion criteria were analysed by an expert flow cytometrist to build the EuroFlow BIgH-IMM database. Data contained in the datafiles was then merged into a reference database that was uploaded in the Infinicyt software (Cytognos, Salamanca, Spain). Subsequently, we compared the results of manual gating (MG) with the performance of two classification algorithms -hierarchical algorithm vs two-step algorithm- for AGI of the cell populations present in 5 randomly selected FCS datafiles. The hierarchical AGI algorithm showed higher correlation values vs conventional MG (r^2^ of 0.94 *vs.* 0.88 for the two-step AGI algorithm) and was further validated in a set of 177 FCS datafiles against conventional expert-based MG. For virtually all identifiable cell populations a highly significant correlation was observed between the two approaches (r^2^>0.81 for 79% of all B-cell populations identified), with a significantly lower median time of analysis per sample (6 *vs.* 40 min, p=0.001) for the AGI tool *vs.* MG, respectively and both intra-sample (median CV of 1.7% *vs*. 10.4% by MG, p<0.001) and inter-expert (median CV of 3.9% *vs*. 17.3% by MG by 2 experts, p<0.001) variability.

**Conclusion:**

Our results show that compared to conventional FC data analysis strategies, the here proposed AGI tool is a faster, more robust, reproducible, and standardized approach for in-depth analysis of B-lymphocyte and PC subsets circulating in human blood.

## Introduction

In recent years, important technological advances have occurred in the field of flow cytometry, which include the development of faster and, highly-accurate multicolour flow cytometers capable of measuring >40 different immunophenotypic markers on tens of thousands of cells per second, together with the availability of a myriad of different (compatible) fluorochrome labels (1–3). Such technological developments have increased our ability for in-depth dissection of hundreds of subsets of human leucocytes, contributing to increase in knowledge about the immune system and its cellular compartments, particularly regarding the T- and B-lymphocyte populations (4–8).

B-cells are key players in the adaptive immune response, where they develop very important roles related to their ability to e.g.: i) mature into plasma cells (PC) capable of producing antigen-specific antibodies of various isotypes and subclasses with multiple immune effector functions (6, 9, 10); ii) generate memory B-cells which can increase (in time and quality) specific immune responses in case of subsequent encounters with an antigen (5, 6); and, iii) act as functional antigen-presenting cells capable of co-stimulating surrounding T-cells (11). Advances in flow cytometry technologies have enabled the identification of >30 subsets of phenotypically and functionally different circulating B-cells in normal blood, which can be further dissected according to the immunoglobulin heavy chain (IgH) isotype and subclass they express (5, 6, 12–14). Of note, the distribution of these distinct maturation-associated functional subsets of B-lymphocytes and PC in blood varies significantly during life, from newborn cord blood to the elderly (5, 6, 10, 15, 16), as well as in different homeostatic states and disease conditions, with important clinical implications (17–24).

At present, multiparameter flow cytometry is the method of choice for fast and high-sensitive identification of such a great number of B-lymphocyte and PC populations that circulate in blood, for both diagnostic and immune monitoring purposes, based on its capacity to simultaneously explore multiple features of high numbers of single cells, via staining with a relatively high number of technically compatible fluorochrome-conjugated antibody reagents (1, 6, 14). Recently, the EuroFlow consortium has designed a new 14-color, 18-antibody combination for in-depth dissection of the B-lymphocyte and PC compartments into >100 cell populations in normal human blood (12, 14). Despite all advantages of such increased analytical capabilities, and the information obtained with it, worldwide usage of the newly-proposed B-cell antibody combination, has been hampered by several limitations associated with current (conventional) manual data analysis procedures. This is because such conventional data analysis approaches are time consuming, hardly reproducible, subjective, and they require high levels of expertise (25–27). Altogether, these features harm standardization and limit implementation of highly informative multicolour (e.g., ≥ 10-color) antibody combinations in routine (flow cytometry) diagnostic practice.

Here we designed, built and validated an innovative, automated, database-guided approach for the evaluation of complex sets of FCS datafiles stained with the EuroFlow BIgH-IMM antibody combination, aimed at fast, standardized and reproducible in-depth dissection of B-lymphocyte and PC subsets in human blood. Our ultimate goal was to provide a tool for a faster, more robust, reproducible and standardized analysis of complex flow cytometry datafiles in routine laboratory diagnosis.

## Materials and methods

### Flow cytometry datafiles used to build the EuroFlow BIgH-IMM database

A total of 36 flow cytometry (FCS) datafiles corresponding to normal peripheral blood (PB) samples collected in EDTA tubes from 17 men and 19 women (median age: 36 years; range 20-62 years) stained with the EuroFlow BIgH-IMM antibody combination were first used for the construction of the EuroFlow BIgH-IMM database. Data contained in these FCS datafiles had been obtained through the measurement of 36 blood samples stained with the EuroFlow BIgH-IMM antibody panel using the EuroFlow standard operating procedures (SOPs) for sample preparation (6), staining of cytoplasmic and cell surface membrane markers (12), instrument calibration, and data acquisition (28), available at (https://euroflow.org). All flow cytometry measurements were performed in LSR Fortessa X20 flow cytometry instruments -Becton/Dickinson Biosciences (BD) San Jose, CA- at four different institutions within the PERISCOPE Consortium (https://periscope-project.eu/consortium): University of Salamanca (USAL; Salamanca, Spain), Leiden University Medical Center (LUMC; Leiden, The Netherlands), Centre for Infectious Disease Control of the National Institute for Public Health and the Environment (RIVM; Bilthoven, The Netherlands), and the University of Turku (UTU; Turku, Finland). For improved identification of PC and their subsets, three samples from pregnant women presenting with an expansion of reactive PC (day +7 to day +10) following vaccination with Boostrix (Glaxo Smith Kline Biologicals PCL., London, UK) were also included in the selected set of FCS datafiles. Each FCS datafile contained information on the 14-color stainings and the light scatter features of a median of 6 x 10^6^ blood nucleated cells (range: 3.7 to 8.8 x 10^6^ cells) obtained from a total volume of 2 ml of lysed whole blood used for the staining of the samples (6). Each stained blood sample was collected after written informed consent had been given by each donor and/or their guardians, according to the Declaration of Helsinki, and the study was approved by the local institutional ethics committees of the four participating centres.

### Construction of the EuroFlow BIgH-IMM database

Selection criteria for the FCS datafiles (out of the 36 FCS datafiles) used to construct the BIgH-IMM database were as follows: i) ≥ 10^5^ cells included in the B-lymphocyte gate defined to include CD19^+^ CD45^+^ forward light scatter (FSC)^lo^/sideward light scatter (SSC)^lo^ single cells; ii) stable flow rate during acquisition as determined in a time vs fluorescence dot plot; iii) appropriate compensation profile according to previously defined criteria (28); and iv) median fluorescence intensity (MFI) distribution for each marker in the corresponding negative and positive control cell populations within the expected predefined range (**Supplementary Table 1**, **Supplementary Figure 1**). Only those FCS datafiles that met all the above criteria were included in the final EuroFlow BIgH-IMM database.

For the analysis of the data contained in each of the 36 FCS datafiles and the construction of the EuroFlow BIgH-IMM database, the Infinicyt software (Cytognos S.L, Salamanca, Spain) was used. Briefly, for database construction, data contained in one randomly selected FCS datafile was first analysed using a clustering algorithm (29) available in Infinicyt. Every cluster of events generated was then assigned to a pre-defined cell population by an experienced flow cytometrist. The first fully analysed (gated) FCS datafile was then stored in Infinicyt and used as the first EuroFlow BIgH-IMM database prototype to subsequently plot the individual clusters of events generated via clustering analysis of data contained in a second FCS datafile. This already provided an automated classification of the individual clusters of events against the (preliminary) database prototype built with the first FCS file, into the cell populations pre-defined in this first FCS database. Then, data contained in the new (second) classified FCS datafile was merged with the data contained in the first classified datafile into a second EuroFlow BIgH-IMM database prototype that contained the pre-classified datafiles 1 plus 2. This second database prototype was subsequently stored in Infinicyt and used for the analysis of the third randomly selected FCS datafile. This process was then repeated until all datafiles that met the selection criteria for inclusion in the database had been processed to build the final EuroFlow BIgH-IMM reference database, ready for its subsequent validation.

### Validation of the EuroFlow BIgH-IMM reference database

For the validation of the EuroFlow BIgH-IMM reference database, a total of 177 additional FCS datafiles (collected in EDTA or Heparin anticoagulated) corresponding to umbilical cord blood (n=15) and PB (n=162) samples -the latter subdivided into 137 healthy donors (74 children aged 7-17, 58 younger adults aged 18-69, and 5 elderly adults aged ≥70 years-old) and 25 patient samples diagnosed with COVID (n=15) or inborn errors of immunity (n=10, including 4 common variable immunodeficiency, 4 selective IgA deficiency, and 2 IgA with IgG subclasses deficiency)- were used, with some healthy samples being post vaccination (28 children obtained at day +7 post-vaccination, 21 children at day +28 post-vaccination, and 10 pregnant women samples studied at day +7 after Boostrix vaccination). Patients with inborn errors of immunity were diagnosed according to the European Society for Immunodeficiencies (ESID) criteria (https://esid.org/Working-Parties/Registry-Working-Party/Diagnosis-criteria). All FCS datafiles in the validation set corresponded to samples that had been stained with the Euroflow BIgH-IMM antibody panel and SOPs in the same four centres involved in generating the datafiles used to build the EuroFlow BIgH database. All FCS datafiles included in the validation set were (blindly) analysed in parallel using: i) manual gating (MG) of the distinct cell populations they contained by an experienced flow cytometrist; and ii) the automated gating and identification (AGI) algorithm and tools implemented in Infinicyt, in combination with the final EuroFlow BIgH-IMM reference database. In a subset of 5/152 datafiles from the validation set of healthy donors, two different automated gating and identification algorithms were tested in parallel, using either a) a two-step sequential algorithm or b) a hierarchical multistep strategy. The two-step sequential algorithm performs an agglomerative clustering of all events, followed by classification of each generated cluster of events into the corresponding cell (debris or doublets) population through direct comparisons against a reference database. The clustering algorithm is based on joining events using multidimensional K neighbours and the Euclidean distances between them, density and affinity calculations, using a K=10 and a 0.9 cut-off (arbitrary units, scaled from 0 to 20) for the discrimination between clusters. Each individual cluster of events identified in the first step, is then classified into a specific population by direct comparison with all reference cell populations in the BIgH-IMM database using canonical analysis (CA; 2-dimensional reduction technique from linear discriminant analysis), as previously described (29). In turn, the hierarchical multi-step approach uses the same algorithms as described above in combination with a pre-defined hierarchical strategy that combines a cell type-specific algorithm based on selection of a given parameter configuration. Accordingly, in this later algorithm, the hierarchical algorithm based on the two-step AGI for the best known parameter configuration for the identification and classification of the major populations of blood leucocytes (eosinophils, neutrophils, monocytes, T/NK-cells, B-cells and other nucleated cells) is first applied. Subsequently, another two-step AGI algorithm is specifically used for further subclassification of the cell populations contained in the B-cell compartment including pre-germinal centre (Pre-GC) B-cells, memory B-cells (MBC) and PC. In the following hierarchical steps, the same algorithm was used except for the classification of the different subsets/maturation stages of Pre-GC (Immature, Naive CD5^+^ and Naive CD5^-^) and PC (CD20^+^CD138^-^, CD20^-^CD138^-^ and CD20^-^CD138^+^), where instead of the two-step sequential AGI algorithm, a maturation algorithm specially designed for the discrimination between groups of cells with heterogeneous antigen expression phenotypes consistent with distinct stages of a maturation pathway (i.e. continuous) based on pre-established cut-offs, was used. The number of events classified within each cell population identified by each algorithm and their corresponding percentage from all leukocytes in the datafile were calculated, recorded, and exported to an excel database for further statistical analyses. Upon comparing the two above-described algorithms, the one that performed the best (more robust analysis or less time consuming in case of similar performance) was selected for the final validation of the AGI approach against manual gating (MG) for the whole set of 177 FCS datafiles in the validation cohort.

### Reproducibility studies

Eight randomly selected datafiles corresponding to PB samples from the validation set of healthy donors were used to determine the intra-sample reproducibility of the MG vs the AGI data analysis approaches. For this purpose, each of these 8 datafiles was analysed five times in parallel by the same expert and the AGI approach. For inter-expert reproducibility, MG and AGI were performed on the same 8 FCS datafiles by two different experts vs the AGI tool. Coefficient of variation (CV) and standard deviation (SD) values were calculated for each cell population identified with both strategies (MG and AGI).

### Statistical analyses

Direct comparison of expert-based MG vs AGI results was performed using the Kruskal-Wallis and Mann-Whitney U tests for paired data. Pearson correlation for normal distributions was used to determine the level of correlation for individual paired data. Statistical comparison of CV data was performed using the paired T test (GraphPad Prism 8.0.2 for Windows; GraphPad Software, San Diego, CA) and the bias between paired measurements was calculated based on Bland-Altman curves using Microsoft Excel 2010 software (version 14; Microsoft Corporation, Albuquerque). For all other statistical analyses, the Statistical Package for Social Sciences (SPSS) software (version 18; IBM, Armonk, NY) was used. Statistical significance was set at p-values ≤.05.

## Results

### Selection of datafiles for inclusion in the EuroFlow BIgH-IMM reference database

**Figure 1:**
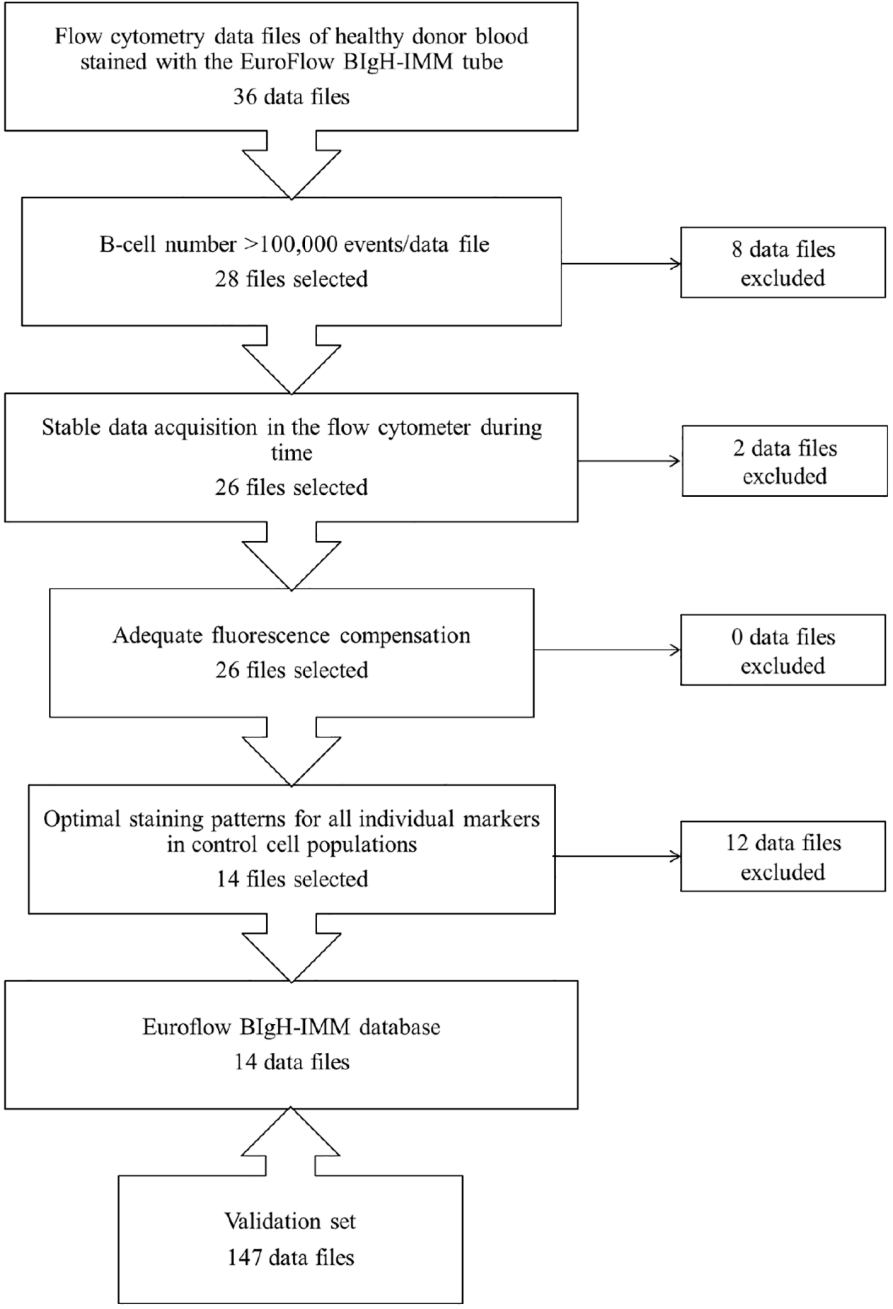
Flowchart illustrating the sequential steps used for the selection of optimal flow cytometry standard (FCS) datafiles to be used to build the EuroFlow BIgH Immune monitoring (IMM) normal blood reference database.

From those 36 PB datafiles stained with the EuroFlow BIgH-IMM antibody panel selected to build the EuroFlow BIgH-IMM database, only 14 (39%) fulfilled all predefined optimal criteria for inclusion in the final reference database (**Figure 1**). In 8/36 (22%) datafiles, the total number of cellular events corresponding to B-lymphocytes and/or PC per datafile was lower than the pre-established threshold of 10^5^ CD19^+^CD45^+^ FSC^lo^/SSC^lo^ B-cell singlets, and thereby, they were excluded from further analyses (**Figure 1**). From the remaining 28/36 datafiles (78%), two showed unstable acquisition of cells during time and they were also excluded (**Figure 1**). All remaining 26/36 datafiles (72%) were then evaluated for appropriate fluorescence compensation, and none was excluded at this step (**Figure 1**). Finally, 12 of the remaining 26 datafiles showed an abnormally high, low and/or heterogeneous staining profile for ≥1 marker evaluated, upon comparison with the corresponding (positive and negative) control cell populations; because of this, these 12 datafiles were also not considered for inclusion in the final database (**Figure 1**). Thus, 14 datafiles were considered to be optimal and selected for inclusion in the final EuroFlow BIgH-IMM database.

### Identification of individual cell populations in the datafiles selected for inclusion in the EuroFlow BIgH-IMM database

**Figure 2:**
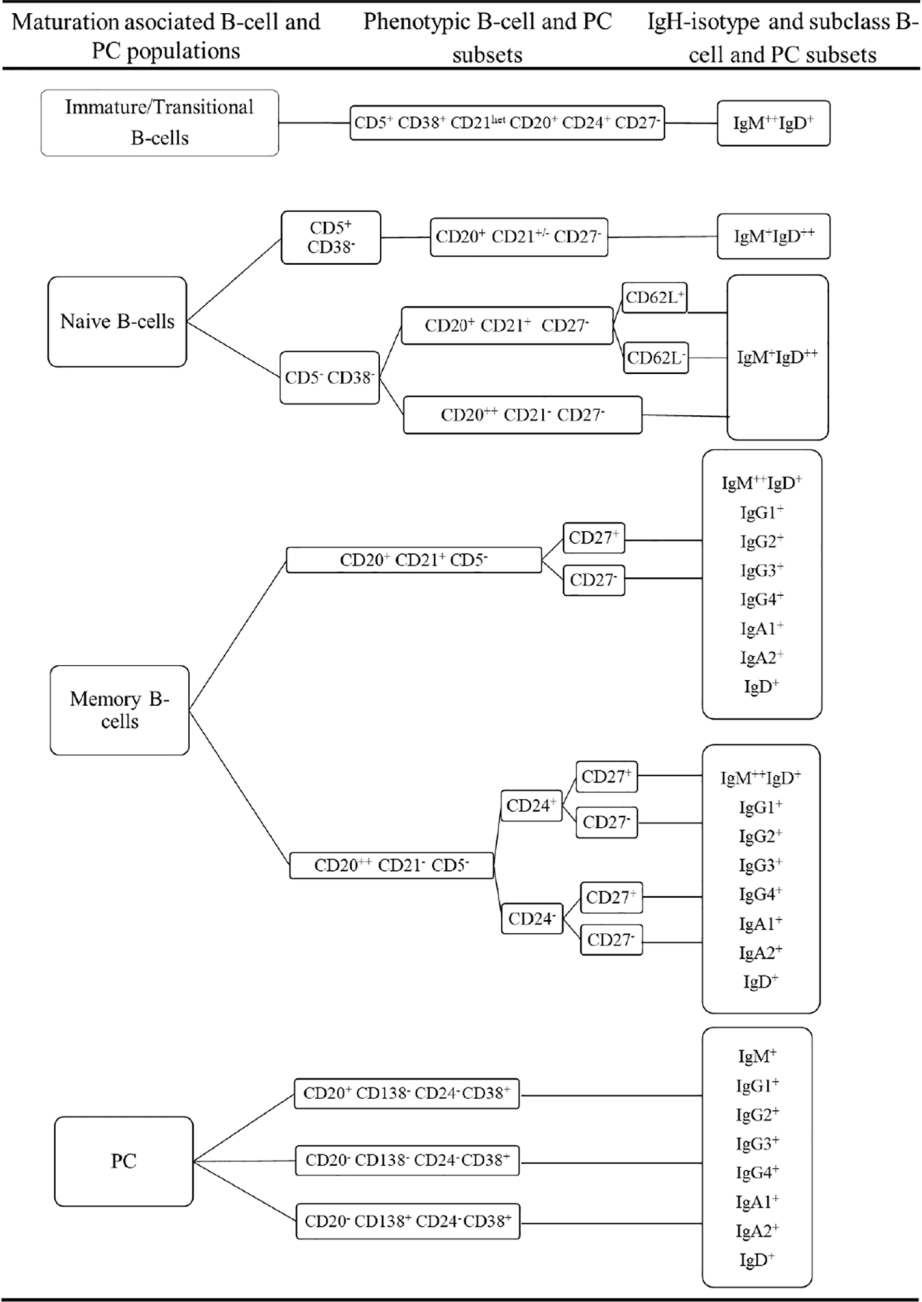
Overall immunophenotypic characteristics of normal circulating blood B-lymphocyte and plasma cell (PC) subsets stained with the EuroFlow BIgH Immune monitoring (IMM) antibody combination.

**Figure 3:**
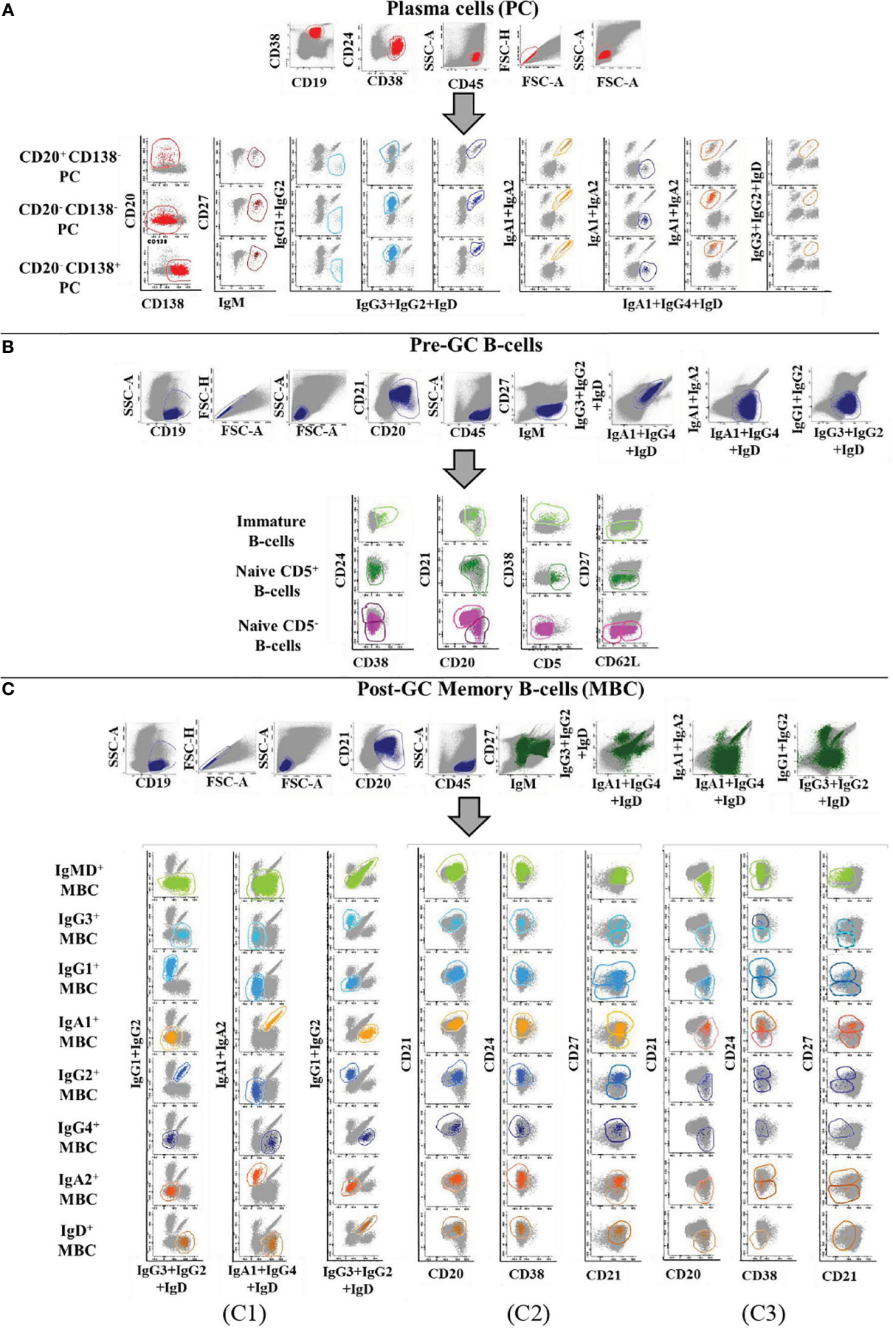
Illustrating example of the Boolean gating strategy used for the identification of the distinct populations of B-lymphocytes and PC circulating in normal human blood upon staining with the Euroflow BIgH-IMM tube, using conventional (manual) gating approaches. B- lymphocytes were defined in **(A-C)** based on their positivity for CD19, after excluding cell debris and doublets. In turn, PC were identified as those events showing a CD38^+^ CD24^-^ CD21^-^ CD45^+^ phenotype, consisting of three maturation-associated subsets (CD20^+^ CD138^-^, CD20^-^ CD138^-^ and CD20^-^ CD138^+^ PC). In **(A)**, each PC subpopulation was classified according to the specific immunoglobulin heavy chain (IgH) isotype and subclass expressed (IgM-D, IgG1-4, IgA1-2, and IgH negative). Within pre-germinal centre (Pre-GC) B-cells, three major subsets of B-lymphocytes were identified: i) immature/transitional B-lymphocytes showing a CD38^+^ CD24^+^ CD21^het^ IgM^hi^ IgD^+^ CD27^-^ CD5^lo^ phenotypic profile; ii) naive CD5^+^ B-lymphocytes showing a CD38^-^ CD24^het^ CD21^+/-^ IgM^+^ IgD^hi^ CD27^-^ CD5^+^ phenotype; and iii) naive CD5^-^ B-lymphocytes showing a CD38^-^ IgM^+^ IgD^hi^ CD27^-^ CD5^-^ phenotype. Subsequently, naive CD5^-^ B-lymphocytes were subclassified according to the pattern of expression of CD21, CD24 and CD62L **(B)**. Finally, memory B-cells (MBC) were identified as those events showing a CD38^-^ CD20^+/++^ CD45^++^ CD5^-^ CD138^-^ phenotype and they were subsequently subdivided according to the IgH^+^ isotype and subclass expressed (C1); the resulting IgH subsets were further subclassified into CD21^+^ CD20^+^ CD27^-/+^ (C2) and CD21^-^ CD20^++^ CD24^-/+^ CD27^-/+^ MBC subsets (C3).

**Figure 4:**
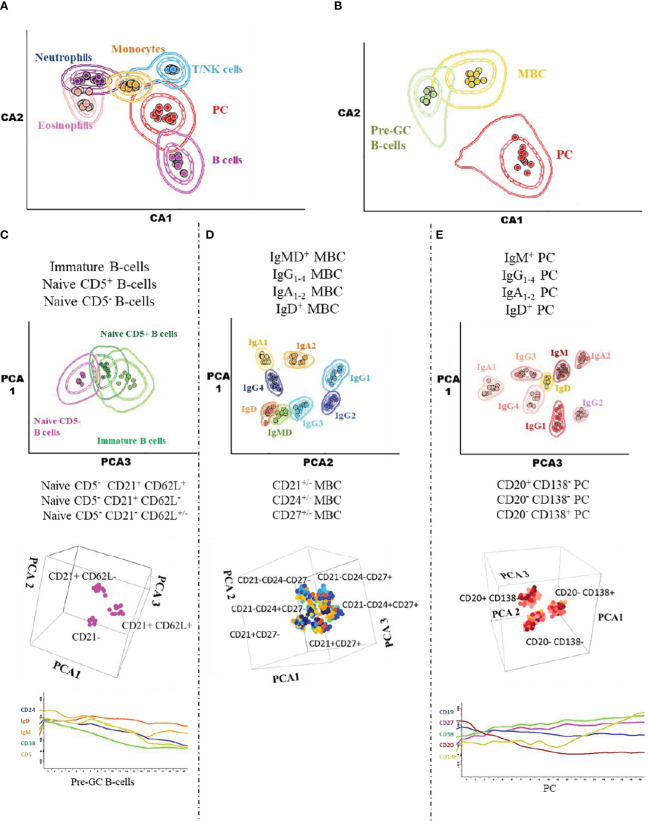
Illustrating 2D-dimensional representation of the multidimensional flow cytometry data contained in the EuroFlow BIgH-IMM database using canonical analysis and principal component analysis graphical plots. The separation achieved in the canonical analysis representations and the principal component analysis plots are shown for the distinct leukocyte **(A)**, total B-cell **(B)**, pre-germinal centre B-cell **(C)**, memory B-cell **(D)** and PC **(E)** populations pre-labelled in the datafiles included in the Euroflow BIgH-IMM reference database of normal human blood via conventional manual gating approaches. Each dot corresponds to the median value for each individual cell population (coded in different colours) in individual datafiles. In the two lower plots in **(C, E)**, the pattern of expression of a group of 5 different markers along the pre-germinal centre and PC maturation stages is shown.

Prior to inclusion in the database, those 14 datafiles that met all the above selection criteria were analysed by an experienced flow cytometrist (AHD) who (manually) gated all identifiable cell populations in each datafile, using the cell type and maturation-associated dendrogram tree profiles illustrated in **Figure 2**. Expert-based manual identification (i.e., manual gating; MG) of individual cell populations present in each FCS datafile selected for further inclusion in the EuroFlow BIgH-IMM database, followed a predefined sequential Boolean gating strategy based on specific combinations of light scatter and immunophenotypic marker expression levels/profiles, as illustrated in **Figure 3**, and described in detail in **Supplementary Text**. Overall, a total of 117 subsets, including 32 different subsets of CD38^hi^ CD19^lo^ CD45^lo^ PC and 83 distinct subsets of pre-GC (n=8) and memory (n=75) CD19^+^ CD45^hi^ FSC^lo^/SSC^lo^ B-lymphocytes were finally identified in these datafiles (**Figures 2**, **3**). All other major (non-PC, non-B-lymphocyte) leucocyte populations were identified based on their different and unique FSC/SSC and immunophenotypic expression profiles with the EuroFlow BIgH-IMM antibody combination. These other major leucocyte populations included: SSC^hi^ CD45^lo^ CD24^+^ neutrophils, SSC^hi^ CD45^+^ CD27^+^ and IgM^+^ autofluorescent eosinophils, SSC^int^ CD45^hi^ monocytes and SSC^lo^ CD45^hi^ CD5^+/-^ T/NK cells, for a total of 123 cell populations (plus cell doublets and debris). Other PB cells that could not be specifically identified (e.g., dendritic cells and basophils) were jointly labelled as “other nucleated cells”. Once all 14 FCS datafiles selected for the database had been manually gated, their contents were merged into the reference EuroFlow BIgH-IMM database, and the resulting merged datafile was uploaded in Infinicyt. For final confirmation of the appropriateness and consistency of expert-based MG, each cell population from each datafile was plotted against the corresponding cell populations from the other datafiles. The results of such comparison were visualized using CA and principal component analysis (PCA) diagrams, where the median and >95% of all events in each population proved to fall within 2 SD of the 2-dimensional CA and PCA plots defined by the same cell population in the other 13 datafiles included in the reference EuroFlow BIgH-IMM database (**Figure 4**).

### Validation of two AGI algorithms vs manual gating

A randomly selected subgroup of 5 FCS datafiles from the 152 datafiles included in the validation set of healthy donors were analysed by an experienced flow cytometrist using MG (please see the text above) and the results obtained were then compared with those of the automated approaches based on the two different AGI algorithms in combination with the reference database. From the 117 B-lymphocyte and PC populations that could be identified, 8 (6.8%) were not reproducibly detected neither by the AGI algorithms nor by the experienced flow cytometrist (i.e., MG). For the other 109/117 B-lymphocyte and PC populations, the hierarchical AGI algorithm showed a significantly better correlation with MG than the two-step AGI algorithm for a total of 28/109 (26%) subsets reproducibly identified in all datafiles, with a median (range) correlation coefficient (r^2^) of 0.865 (0.03-1.00) (p<0.8) vs 0.59 (0.019-0.76), (p<0.83), respectively. Even if some subsets 10/28 showed an overall lower correlation (r^2^ < 0.81) against MG, the hierarchical AGI algorithm was still able to identify them, in contrast to the two-step AGI algorithm. In contrast, the two-step AGI algorithm showed a better performance than the hierarchical algorithm in only 5/109 cell populations (5%) with a median r^2^ (range) of 0.884 (0.814-0.974) (p<0.036) vs 0.271 (< 0.001-0.64) (p<0.97), respectively. For the remaining 76/109 populations, similarly high median correlation levels were observed for the hierarchical and two-step AGI algorithms vs expert-based MG (r^2^ of 0.974 vs 0.964; range: <0.001-1.00 vs <0.001-1.00, respectively) (**Supplementary Table 2**). Importantly, the hierarchical AGI algorithm required almost half of the computer calculation time needed by the two-step AGI algorithm with a median (range) time of analysis per datafile of 6 (5–7) vs 11 (9–13) min (p=0.001). Based on these results, the hierarchical AGI algorithm was selected, and subsequently used for the validation of the EuroFlow BIgH-IMM reference database on the independent whole validation set of 152 healthy donors and 25 patients FCS datafiles.

### Validation of the EuroFlow BIgH-IMM reference database

**Figure 5:**
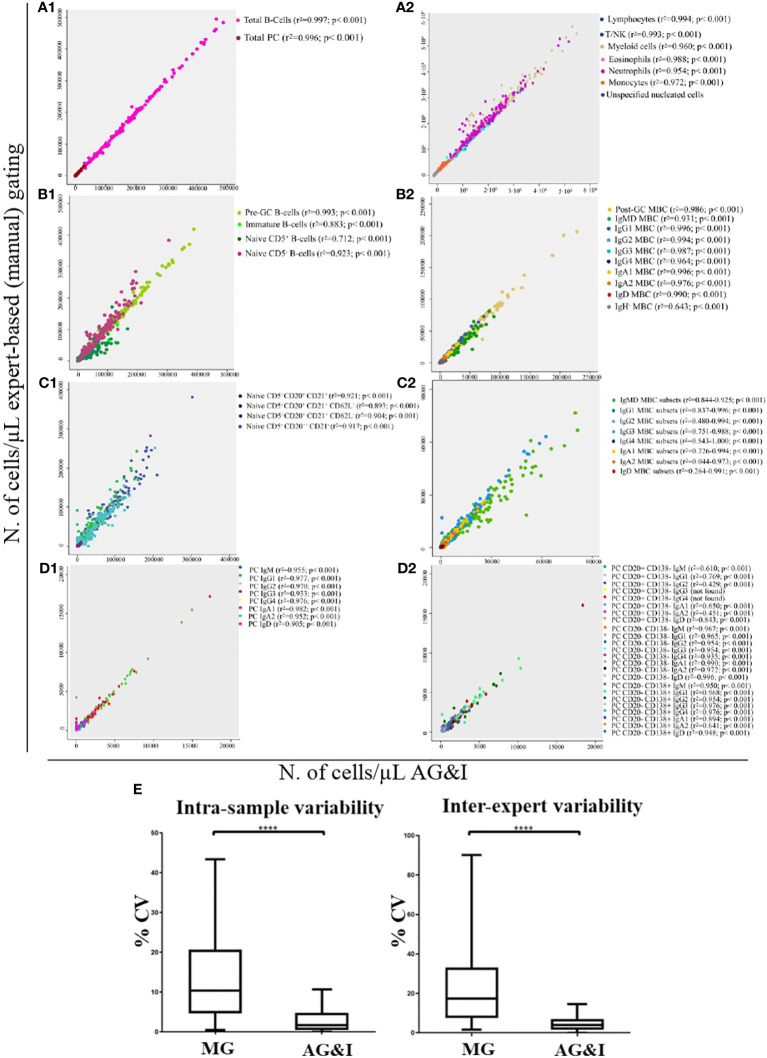
Correlation between the number of events classified as belonging to the different immune cell populations present in normal peripheral blood samples as analysed by expert-based manual gating vs the AGI tool. **(A1)**, total B-lymphocyte cells (r^2 = ^0.997; p< 0.001) and total PC (r^2 = ^0.996; p< 0.001); **(A2)**, other major non-B/non-PC leukocyte populations; **(B1)**, major pre-GC B-cell subsets; **(B2)**, major memory B-cell (MBC) subsets; **(C1)**, minor pre-GC B-cell populations; **(C2)**, minor MBC populations; **(D1)**, major PC subsets and **(D2)**, minor PC populations. In **(E)**, intra-assay and inter-expert reproducibility of MG and AGI. Box-and-Whisker plots of CVs (%) for all lymphoid populations. ^****^Statistically significantly different (p< 0.0001) based on paired t-Test.

Using the batch analysis function of Infinicyt and the EuroFlow BIgH-IMM database, each of the 152 FCS datafiles included in the validation set of healthy donors was analysed and checked by an expert who classified all events into their corresponding 123 normal (or debris/doublets) cell populations (of which 3 were found to be absent in this validation set). For each individual case, the number of events classified in each cell population and their frequencies were compared with the results obtained via (blinded) MG by the same expert in the same (anonymized) FCS datafiles. Total B-lymphocyte and total PC populations analysed with the AGI algorithm showed a median frequency (range) among all nucleated cells present in individual blood samples of 4.9% (0.5%-13%) and 0.06% (<0.001%-0.8%) vs 4.5% (0.5%-12%) and 0.05% (<0.001%-0.8%) as obtained by MG (p< 0.001), respectively. Overall, for the great majority (97/123, 79%) of the quantifiable (>50 cells identified, except for >20 cells for PC subsets) cell populations with the EuroFlow BIgH-IMM antibody panel, a high statistically significant (p<0.001) degree of correlation (r^2^> 0.81) -median (range): 0.958 (0.810-1)- was observed between the AGI and MG approaches. These included total B-lymphocytes and PC, as well as other major non-PC and non-B-lymphocyte populations (8/8), including 12/14 major B-lymphocyte subsets, and 8/8 major PC subsets, with a median (range) correlation coefficient (r^2^) of 0.991 (0.956-0.997) (p<0.001), 0.987 (0.883 - 0.996) (p< 0.001) and 0.963 (0.905-0.982) (p<0.001), associated with a high degree of agreement (<10% to <20% discordant values), respectively (**Figure 5**, A1-2, B1-2, D1). Similarly, a high degree of correlation between the AGI and MG was also obtained for most minor populations of B-lymphocytes - 51/69, (74%) with median r^2^ values (range) of 0.918 (0.810 to 1.000) (p< 0.001)-, and PC subsets -18/24, (75%) with median r^2^ values (range) of 0.960 (0.843-0.996) (p<0.001)- (**Figure 5**, C1-2, D2). For the remaining B-lymphocyte and PC subsets acceptable median correlation (r^2^) levels were found of 0.543 (p<0.001) and 0.646 (p<0.001), respectively (**Supplementary Table 3**).

In a subsequent phase of the study aimed at demonstrating the reliability of this approach, two additional analyses were done for validation purposes. Firstly, a validation across various age groups was done, where separate analyses were performed for each age group: i) healthy children (n=15); ii) healthy adults (n=15); and iii) healthy elderly adults (n=5), using selected samples from the previous healthy donor cohort. The great majority -101/123 (82%), 95/123 (77%) and 108/123 (88%), respectively- of quantifiable (>50 cells identified, except for >20 cells for PC subsets) cell populations showed a high (statistically significant) degree of correlation, with median r^2^ values (range) of 0.980 (0.835-1.000) (p< 0.001), 0.952 (0.829-1) (p< 0.001) and 0.995 (0.814-1), respectively. In the second validation set of analysis, data corresponding to COVID-19 patients (n=15) and immunodeficiency patients (n=10) were specifically analyzed. Likewise, a high degree of correlation between the AGI and MG was also obtained, for the great majority -99/123 (80%)- of the quantifiable cell populations with median r^2^ values (range) of 0.961 (0.832-1) (p<0.001).

Based on a standard computer - Intel Core™ i7-4720HQ CPU @ 2.60GHz 16-64GB RAM; operative system of 64bits - the AGI approach required less time than MG, for the analysis of each individual FCS datafile, with a median (range) time of analysis of 6 min (5-7 min) vs 40 min (30-50 min), respectively (p=0.001).

### Intra-sample and inter-expert reproducibility

The intra-sample and inter-expert reproducibility of the AGI tool vs MG was evaluated through the analysis of replicates of the same blood samples by the same expert and by two different experts (vs AGI), respectively. Overall, the AGI- based approach showed a significantly lower median CV compared with MG for both intra-sample and inter-expert reproducibility analyses: 1.7% vs 10.4% (p<0.001) and 3.9% vs 17.3% (p<0.001), respectively (**Figure 5E**).

## Discussion

In recent years, technological advances in multicolour flow cytometry have expanded our ability to dissect in an unprecedented depth, the different compartments of immune cells present in blood and other body fluids and tissues, with the ability to identify a significantly broader number of leucocyte populations, particularly among T-lymphocytes and B-cells (6, 7, 30–32). This has led to an exponential growth of the amount of data generated from a single aliquot of a relatively small volume of a sample (e.g., blood). Such technological advances have thereby contributed to an increase in our knowledge about immune cells in both homeostatic and disease conditions, due to the enhanced capability of measuring millions of cells stained with dozens of markers simultaneously (33–36). However, new challenges in flow cytometry data analysis have also emerged. As these new flow cytometry datafiles contain data on millions of cells for dozens of different cell populations, the required time and expertise to analyse these files, based on conventional 2-D dot plot-based MG strategies (locally defined), have increased exponentially (7, 37). Importantly, the use of such conventional expert-based MG (i.e., data analysis) approaches for the analysis of FCS data sets containing high numbers of identifiable cell populations, would also lead to higher levels of subjectivity, and thereby, to a decreased reproducibility among experts, hampering robust exchange of data in multicentric studies (7, 37).

Here, we designed, built, and validated a new, fast and highly reproducible approach for automated gating and identification of >100 different immune cell (i.e., B- lymphocyte and PC) populations in human blood, using the EuroFlow BIgH-IMM 18-antibody combination as a model. The panel was designed to dissect the B-cell compartment based only on phenotypic grounds, leaving aside further functional analyses. Due to the availability of new flow cytometers capable of measuring >40 different markers simultaneously, future versions of the EuroFlow BIgH-IMM antibody combination could incorporate new functional markers (such as cell activation, exhaustion, memory, transcription factors, and cytokines), for an (even more) in-depth parallel phenotypic and functional assessment of the B-cell and PC stage compartments. The new AGI approach relies on the combined use of a clustering algorithm and, for the first time, a reference database of high-quality FCS datafiles generated in four different centres based on a standardized approach, aimed at the identification of clusters of events and their objective classification in up to 123 blood cell populations (5, 6, 10), with different functional roles in the immune response. Of note, the AGI approach allowed for the robust and more reproducible identification of virtually all (>100) groups of blood cells present in a multidimensional space (n=18 dimensions) compared to conventional MG approaches based on 2-D sequential Boolean gating strategies (38–40). In addition, this new AGI approach has several important advantages compared to other previously reported software-based automated data analysis approaches. Thus, i) it does not need pre-processing of the datafiles with an external software (e.g. with tools from FlowCore) (41); ii) the hierarchical clustering strategy proposed enables combined use of different algorithms and configurations for the definition of distinct cell populations, a novel concept compared to those used by other previously reported approaches such as SPADE (42), FlowSOM (43) or Citrus (44), that are solely based on the use of a single algorithm for the identification of single cell populations across all cell compartments in a sample; iii) it takes advantage of using a reference database for classification of individual clusters of events instead of sequential gating or standing alone clustering algorithms (e.g. AutoGate (38) or Cytometree (45)), which provides more objective and reproducible identification of the multiple cell populations contained in a sample including both major and minor immune cell populations; and, iv) it automatically classifies all individual clusters of events into the different well-defined immune cell compartments present in a sample, which are mirrored in the previously defined related cell populations in the database, which is a critically relevant feature in the clinical settings for the specific identification of abnormal (e.g., clonal and/or phenotypically aberrant) cell populations. This later feature is also unique to the here proposed AGI approach vs other previously described algorithms and software tools such as FlowSOM, SPADE, Citrus, PhenoGraph or FLOCK that group the clusters of events solely based on combinations of markers (46–48).

To reach our objectives, we first built a database of normal blood stained with a single 14-color, 18-antibody combination previously designed by the EuroFlow consortium (14), which allows simultaneous identification and characterization of 117 B- lymphocyte and PC populations (5, 6, 10). To build the EuroFlow BIgH-IMM database, strict criteria were followed in selecting individual datafiles, aiming at ensuring minimum technical mistakes while keeping intrinsic instrument and both intra- and inter-laboratory daily variability. For optimal performance, only those healthy donor/reactive datafiles containing a minimum of 100,000 B-lymphocyte and PC numbers, with optimal staining profiles for each of the 18 antibodies used to stain the samples, were used to build the database. The threshold for the B-cell counts in the healthy donor/reactive cohort was established considering that there are minor B-cell subsets that would be unquantifiable at low sensitivity levels, these files being used for validation purposes. The B-cell count threshold was not applied to the smaller patient cohort, which is thereby, representative of routine laboratory diagnostic settings. Subsequently, in each of the selected datafiles, all immune cell populations and their subsets were identified by an expert flow cytometrist based on conventional MG procedures. As expected, the patterns of expression of CD38 and CD20 together with CD19, CD45 and light scatter allowed specific identification of CD45^hi^ CD19^+^ CD20^hi^ CD38^-/lo^ FSC^lo^/SSC^lo^ B-lymphocytes and CD45^lo^ CD19^lo^ CD20^-/lo^ CD38^+^ FSC/SSC^int^ PC. Further, staining for CD138 and CD20 provided the basis to split PC into three different maturation-associated subsets: i) the more immature CD138^-^ CD20^+^ PC; the ii) intermediate CD138^-^ CD20^-^ PC; and iii) the more mature CD138^+^ CD20^-^ PC (5). Each of these three PC subsets could be further subclassified according to the specific IgH isotype and subclass expressed into a total of 32 distinct PC populations. Despite this extended PC subsetting using the 13 PC-specific markers, a population was considered to be quantifiable only when at least 20 events assigned to it. Likewise, B-lymphocytes were also divided into pre-GC antigen-unexperienced B-lymphocytes and post-GC antigen-experienced MBC, which were further subdivided into a total of 85 B-lymphocyte subsets based on the IgH isotype and subclass expressed, and the pattern of expression of the CD27, CD21, CD5, CD24, CD62L and CD38 cell surface markers. Briefly, Pre-GC cells were subdivided into three major populations of immature, CD5^+^ naive and CD5^-^ naive B-lymphocytes, while MBC were subdivided into subsets of B-lymphocytes expressing different IgH isotypes and subclasses, each of which was further split into additional minor subsets based on their pattern of expression (positive vs negative) of CD27, CD24 and CD21. For the entire B-cell compartment (pre-GC B-cells, MBC and PC), we identified a total of 117 different subsets, from which 116 were found to be actually present in the blood samples included in the database (absent subsets could be donor-dependent and/or the number of cells <50). Once expert-based MG of the datafiles selected for the reference database had been completed, the analysed FCS datafiles were merged into a single datafile which was uploaded in the Infinicyt software as the EuroFlow BIgH-IMM reference database.

Once (the building of) the reference database had been completed, it was used to evaluate the performance of two different (hierarchical AGI and two-step AGI) algorithms for automated gating and identification of the different cell populations identifiable in human blood with the EuroFlow BIgH-IMM antibody panel. This was attained through direct comparison of each individual cluster of events identified in individual blood samples with the two AGI algorithms, against each of the pre-defined cell populations in the reference database, with the results obtained by MG performed by an experienced flow cytometrist (7, 30, 33, 37, 49, 50). From the two AGI algorithms evaluated, the hierarchical algorithm, based on *a priori* expert knowledge about the most relevant parameters for identification of specific cell populations in combination with the two-step AGI algorithms and pre-defined cut-offs for reproducible discrimination within heterogeneous (continuous) cell populations of their distinct maturation stages, proved to provide more accurate immune cell subsetting than the two-step AGI algorithm on its own, taking MG as a reference. In addition, the hierarchical algorithm also proved to be significantly faster than both the two-step AGI algorithm and particularly, the expert-based MG approach. Based on these results the hierarchical clustering algorithm was selected for further validation of the database-guided AGI tool against the conventional expert-based MG approach in a large validation cohort of (out-of-database sample) FCS datafiles of normal blood stained with the EuroFlow BIgH-IMM antibody combination.

Overall, the results obtained with the new AGI approach showed a high degree of correlation with MG data, as regards the identification and enumeration of the great majority of B-lymphocyte, PC and non-B-lymphocyte/non-PC populations identifiable in human blood with the EuroFlow BIgH-IMM antibody panel, independently of age or specific clinical conditions (healthy, viral infection or immunodeficiency) of the donor. In addition, these results suggest that the use of a high number of markers to define individual populations in the higher multidimensional marker-space as performed automatically by the AGI software tool in this study, also mitigates any potential (physiological or pathological) modulation of cell surface membrane or intracellular expression levels of individual molecules, as there were no significant/relevant differences in its performance vs manual data analysis. Importantly, the few differences observed were restricted to cell populations, which were rarely represented in the datafiles used for validation purposes, hampering their unequivocal identification by both the expert and the AGI software approaches (51). In addition, the new AGI tool proved to be a more robust approach for reproducible data analysis, since it was associated with significantly lower levels of variability and more reproducible results vs conventional MG, both at the intra-sample and the inter-expert levels. Altogether, these results make the new here-described automated gating tool particularly attractive for multicentric studies where comparison of large sets of flow cytometry data obtained in different laboratories, is required. Another remarkable advantage of the automated gating tool here proposed (vs conventional MG), relies on the time required for the analysis of individual (or a set of) datafiles, since the former approach speeded up data analysis by reducing the processing time substantially, (>80%) when compared to conventional MG.

In summary, here we designed, constructed and validated a new database and hierarchical clustering algorithm for faster and more robust, reproducible and standardized automated gating and identification of large numbers of B-lymphocyte and PC populations in human blood, suitable for detailed monitoring of immune responses after vaccination and fast characterization of B-cell and PC alterations in multiple disease conditions such as infection, autoimmunity, primary and secondary immunodeficiency both at diagnosis and after therapy within individual laboratories and in multicentric clinical studies. An illustrative example would be the use of the proposed approach for the diagnosis and classification of predominantly antibody deficiencies such as common variable immunodeficiency, combined immunoglobulin isotype deficiency and selective IgA deficiency, respectively (17).

## Data availability statement

The raw data supporting the conclusions of this article will be made available by the authors, without undue reservation.

## Ethics statement

The studies involving humans were approved by Complejo Asistencial Universitario de Salamanca. The studies were conducted in accordance with the local legislation and institutional requirements. The participants provided their written informed consent to participate in this study.

## Author contributions

AH-D: Writing – original draft, Writing – review & editing. RF: Writing – review & editing. MP-A: Writing – review & editing. AMD: Writing – review & editing. JG-B: Writing – review & editing. A-MB: Writing – review & editing. EB: Writing – review & editing. AT-V: Writing – review & editing. MB: Writing – review & editing. GG: Writing – review & editing. JvD: Writing – review & editing. AO: Writing – original draft, Writing – review & editing.
