# Supplementary material for: Automated EuroFlow approach for standardized in-depth dissection of human circulating B-cells and plasma cells
Source: Front Immunol. 2023 Oct 17;14:1268686. doi: 10.3389/fimmu.2023.1268686 (PMC10616957; doi:10.3389/fimmu.2023.1268686)
Supplement: Supplementary file 1 [file DataSheet_1.docx]

**Supplementary materials:**

**Supplementary text.**

Circulating PC were first identified based on their positivity for CD38^hi^ CD19^lo^ CD45^lo^ in the absence of CD24, and their intermediate forward (FSC) and sideward (SSC) light scatter properties. Then, debris and doublets (e.g., events with lower FSC-Area vs SSC-Area and/or events placed outside the FSC-Area vs FSC-Height dotplot diagonal) were excluded from the PC gate, while the remaining cellular events were selected and assigned as PC. PC were then subdivided into three subsets based on their maturation stage-associated phenotypes as defined by their expression profile for CD20 and CD138: i) CD20^+^ CD138^-^; ii) CD20^-^ CD138^-^; and iii) CD20^-^ CD138^+^ PC. Each of these three maturation-associated PC subsets was subsequently subclassified according to the specific IgH isotype and IgH subclass expressed (Figure 3A) for a total of 32 different PC subsets (Figures 2 and 3). Afterward, B-lymphocytes were gated based on their positivity for CD19, strong staining for CD45^hi^ and FSC^lo^/SSC^lo^ features. Cell debris and doublets contained in the B-lymphocyte gate were then excluded, as described above for PC. Pre-GC B-lymphocytes were identified as CD138^-^ CD27^-^ IgMD-double positive B-cells, and memory B-cells (MBC) were identified among all B-cell singlets as those cellular events displaying a CD20^+/hi^ CD45^hi^ CD5^-^ and CD38^-^ immunophenotype after excluding pre-GC B-cells. Then, CD27^-^ pre-GC B-cells were further subdivided into i) immature CD5^+^ CD38^+^ CD21^het^ CD20^+^ CD24^+^ IgM^++^ IgD^+^ B-lymphocytes, ii) naïve CD5^+^ CD38^-^ CD21^+/-^ CD20^+^ CD24^+^ IgM^+^ IgD^++^, iii) naïve CD5^-^ CD38^-^ CD21^+^ CD20^+^ CD62L^+^ IgM^+^ IgD^++^, iv) naïve CD5^-^ CD38^-^ CD21^+^ CD20^+^ CD62L^-^ IgM^+^ IgD^++^ and v) naïve CD5^-^ CD38^-^ CD21^-^ CD20^+^ IgM^+^ IgD^++^ B-cells (Figure 3B). Based on the pattern of expression of the different IgH isotypes and subclasses (Figure 3C1) and the expression profile for CD20, CD21, and CD24 (Figure 3C2-3), MBC were also subdivided into: i) CD27+ unswitched IgMD+ MBC, including CD5^-^ CD38^-^ CD21^+^ CD20^+^ IgM^+^ IgD^+^, CD5^-^ CD38^-^ CD21^-^ CD20^+^ CD24^+^ IgM^+^ IgD^+^ and CD5^-^ CD38^-^ CD21^-^ CD20^+^ CD24^-^ IgM^+^ IgD^+^ subsets and ii) switched MBC which were further subsetted on the basis of the IgH expressed into IgG_1-4_, IgA_1-2_ and IgD^+^ and their corresponding CD20, CD21, CD24 and CD27 positive vs negative subsets (Figure 3C).

**Supplementary Table 1.** Cell populations used as negative and positive controls for the evaluation of median fluorescence intensity values obtained for each marker conjugated with a given fluorochrome as stained in the data files used for the construction of the EuroFlow BIgH-IMM database (n=14).

| **Marker-fluorochrome** | | **Positive Control** | | **Negative Control** | | **N. of data files with out of range values** |
| --- | --- | --- | --- | --- | --- | --- |
| CD19-BV786 | | B-cells: | 4988 (3555-8040) | T/NK: | 54 (26-118) | 0/36 (0%) |
| CD20-PECF594 | | B-cells: | 6456 (4000-13,000) | T/NK: | 21 (7-94) | 12/36 (33%) |
| CD21-BV711 | | B-cells: | 4465 (2875-5511) | T/NK: | 168 (17-351) | 0/36 (0%) |
| CD45-AF700 | | B-cells: | 14022 (11229-19408) | NA |  | 0/36 (0%) |
| CD27-BV421 | | B-cells CD27+: | 1672 (1229-2456) | B-cells CD27-: | 152 (84-231) | 0/36 (0%) |
| CD24-BV650 | | Neutrophils: | 4091 (2557-6131) | T/NK: | 249 (111-416) | 0/36 (0%) |
| CD5-PECy7 | | T-cells: | 32232 (20725-35025) | MBC: | 503 (215-1035) | 0/36 (0%) |
| CD138-PECy7 | | PC CD138+: | 18311 (16137-21563) | PC CD138-: | 750 (167-2886) | 0/36 (0%) |
| IgM-BV510 | | Naive B-cells: | 2755 (2300-5352) | T/NK: | 274 (232-369) | 0/36 (0%) |
| IgD | -FITC+APC | Naive B-cells: Naive B-cells: | 3596 (3342-5091)  5617 (4455-7458) | T/NK:  T/NK: | 602 (502-706)  349 (292-520) | 0/36 (0%) |
| CD38-APCH7 | | PC: | 15904 (10000-20000) | MBC: | 99 (23-175) | 0/36 (0%) |
| IgG1-PE | | MBC IgG1: | 21009 (15740-32473) | T/NK: | 218 (22-412) | 0/36 (0%) |
| IgG2 | -PE+FITC | MBC IgG2: MBC IgG2: | 10757 (7840-11486)  6096 (4984-6811) | T/NK:  T/NK: | 218 (22-412)  633 (502-706) | 0/36 (0%) |
| IgG3-FITC | | MBC IgG3: | 6884 (6214-9150) | T/NK: | 633 (502-706) | 0/36 (0%) |
| IgG4-APC | | MBC IgG4: | 9661 (7517-13749) | T/NK: | 332 (292-520) | 0/36 (0%) |
| IgA1 | -PerCPCy5.5+APC | MBC IgA1: MBC IgA1: | 7012 (3241-9660)  16309 (11905-21359) | T/NK:  T/NK: | 146 (26-300)  332 (292-520) | 0/36 (0%) |
| IgA2-PerCPCY5.5 | | MBC IgA2: | 5422 (3044-7809) | T/NK: | 146 (26-300) | 0/36 (0%) |

^*^ Results expressed as MFI (median fluorescence intensity; arbitrary fluorescence units scaled from 0 to 100,000). BV- Brilliant Violet; PE-Phycoerythrin; AF-Alexa Fluor; Cy-Cyanine; FITC-Fluorescein isothiocyanate; APC-Allophycocyanin; PerCP-Peridinin Chlorophyll.

**
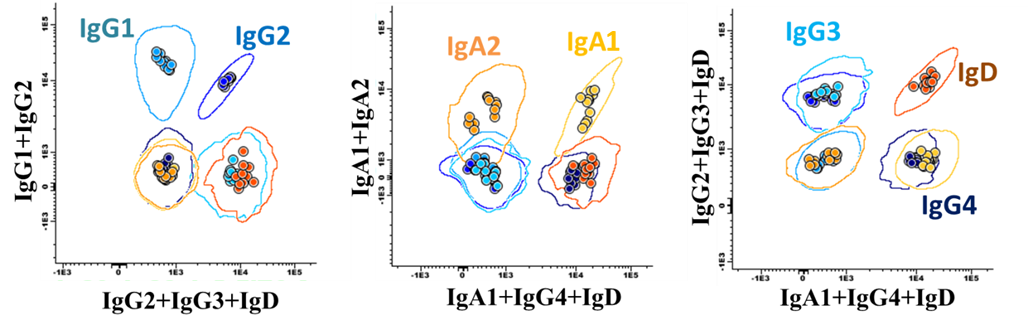
**

**Supplementary Figure 1**. Reference image used for the evaluation of the B-cell and plasma cell subsets expressing different IgH isotypes and subclasses included in the data files used for the construction of the EuroFlow BIgH-IMM database.

**Supplementary Table 2-** Comparison between the two AG&I algorithms used in combination with the EuroFlow BIgH-IMM reference database vs expert-based manual gating for the analysis of randomly selected data files (n=5) stained with the EuroFlow BIgH-IMM antibody combination.

| **Cell populations** | **Manual analysis** | | **AG&I Algorithm1** | | **AG&I Algorithm2** | | **AG&I Algorithm1 vs MA** | **AG&I Algorithm2 vs MA** |
| --- | --- | --- | --- | --- | --- | --- | --- | --- |
| B-lymphocytes | 4.1 | (2.4-5.3) | 4.7 | (2.6-5.7) | 4.5 | (2.5-5.6) | 0.998 (p<0.001) | 0.992 (p<0.001) |
| Pre-GC B cells | 2.8 | (1.3-3.6) | 3.3 | (1.5-4.2) | 3.1 | (1.4-4.1) | 1.000 (p<0.001) | 0.990 (p<0.001) |
| Immature B cells | 0.2 | (0.04-0.2) | 0.3 | (0.08-0.4) | 0.09 | (0.02-0.2) | 0.940 (p=0.005) | 0.563 (p=0.144) |
| Naive CD5+ B cells | 0.4 | (0.09-0.6) | 0.2 | (0.01-0.4) | 0.8 | (0.2-1.4) | 0.640 (p=0.100) | 0.720 (p=0.070) |
| Naïve B cells | 2.2 | (1.2-2.9) | 2.7 | (1.4-3.6) | 2.2 | (1.2-2.7) | 0.972 (p=0.002) | 0.930 (p=0.008) |
| Naive B CD20+CD21+ | 2.2 | (1.2-2.9) | 2.7 | (1.4-3.6) | 2.1 | (1.1-2.7) | 0.976 (p=0.002) | 0.927 (p=0.009) |
| Naive B CD20+CD21+CD62L+ | 2 | (1.1-2.7) | 2.3 | (1.2-3.2) | 1.9 | (1-2.5) | 0.972 (p=0.006) | 0.977 (p=0.004) |
| Naive B CD20+CD21+CD62L- | 0.2 | (0.1-0.4) | 0.3 | (0.2-0.5) | 0.3 | (0.08-0.9) | 0.416 (p=0.240) | 0.955 (p=0.008) |
| Naive B CD20++CD21- | 0.04 | (0.02-0.08) | 0.0 | (0.02-0.08) | 0.04 | (0.02-0.08) | 0.962 (p=0.003) | 0.865 (p=0.022) |
| Memory B-cells (MBC) | 1.3 | (0.5-2.6) | 1.4 | (0.6-2.7) | 1.4 | (0.6-2.7) | 1.000 (p<0.001) | 0.994 (p<0.001) |
| MBC IgMD+ | 0.6 | (0.3-1.5) | 0.7 | (0.4-1.6) | 0.7 | (0.4-1.6) | 0.998 (p<0.001) | 0.994 (p<0.001) |
| MBC IgMD CD20+CD21+ | 0.6 | (0.3-1.4) | 0.6 | (0.4-1.5) | 0.6 | (0.3-1.5) | 0.998 (p<0.001) | 0.994 (p<0.001) |
| MBC IgMD CD20++CD21- | 0.05 | (0.02-0.1) | 0.03 | (0.000-0.07) | 0.06 | (0.02-0.1) | 0.872 (p=0.020) | 0.996 (p<0.001) |
| MBC IgMD CD20++CD21-CD24+ | 0.05 | (0.02-0.1) | 0.03 | (0.000-0.07) | 0.06 | (0.02-0.1) | 0.867 (p=0.022) | 0.994 (p<0.001) |
| MBC IgMD CD20++CD21-CD24- | 0.004 | (0.001-0.007) | 0.002 | (0.000-0.004) | 0.003 | (0.000-0.005) | 0.865 (p=0.022) | 0.094 (p=0.613) |
| MBC IgG1+ | 0.3 | (0.1-0.4) | 0.3 | (0.1-0.4) | 0.3 | (0.1-0.4) | 0.998 (p<0.001) | 0.996 (p<0.001) |
| MBC IgG1 CD20+CD21+ | 0.2 | (0.08-0.3) | 0.3 | (0.09-0.4) | 0.2 | (0.09-0.3) | 1.000 (p<0.001) | 0.998 (p<0.001) |
| MBC IgG1 CD20+CD21+CD27+ | 0.2 | (0.06-0.3) | 0.2 | (0.07-0.3) | 0.2 | (0.07-0.3) | 0.951 (p=0.005) | 0.992 (p<0.001) |
| MBC IgG1 CD20+CD21+CD27- | 0.04 | (0.02-0.06) | 0.04 | (0.0002-0.08) | 0.06 | (0.02-0.09) | 0.610 (p=0.119) | 0.912 (p=0.011) |
| MBC IgG1 CD20++CD21- | 0.04 | (0.01-0.07) | 0.04 | (0.01-0.06) | 0.05 | (0.02-0.08) | 0.970 (p=0.002) | 0.956 (p=0.004) |
| MBC IgG1 CD20++CD21-CD24+ | 0.02 | (0.006-0.04) | 0.008 | (0.001-0.02) | 0.02 | (0.01-0.05) | 0.968 (p=0.002) | 0.925 (p=0.009) |
| MBC IgG1 CD20++CD21-CD24+CD27+ | 0.01 | (0.003-0.02) | 0.003 | (0.000-0.006) | 0.01 | (0.007-0.03) | 0.604 (p=0.122) | 0.856 (p=0.025) |
| MBC IgG1 CD20++CD21-CD24+CD27- | 0.007 | (0.002-0.01) | 0.005 | (0.001-0.01) | 0.008 | (0.002-0.02) | 0.982 (p=0.001) | 0.982 (p=0.001) |
| MBC IgG1 CD20++CD21-CD24- | 0.02 | (0.008-0.04) | 0.03 | (0.01-0.05) | 0.02 | (0.008-0.04) | 0.980 (p=0.001) | 0.988 (p=0.001) |
| MBC IgG1 CD20++CD21-CD24-CD27+ | 0.004 | (0.001-0.007) | 0.002 | (0.000-0.004) | 0.005 | (0.002-0.009) | 0.591 (p=0.129) | 0.984 (p=0.001) |
| MBC IgG1 CD20++CD21-CD24-CD27- | 0.02 | (0.006-0.03) | 0.03 | (0.01-0.05) | 0.02 | (0.007-0.03) | 0.982 (p=0,001) | 0.982 (p=0.001) |
| MBC IgG2+ | 0.09 | (0.02-0.2) | 0.1 | (0.02-0.2) | 0.1 | (0.03-0.2) | 0.998 (p<0.001) | 0.998 (p<0.001) |
| MBC IgG2 CD20+CD21+ | 0.09 | (0.02-0.1) | 0.1 | (0.02-0.2) | 0.1 | (0.03-0.2) | 1.000 (p<0.001) | 1.000 (p<0.001) |
| MBC IgG2 CD20+CD21+CD27+ | 0.08 | (0.02-0.1) | 0.1 | (0.02-0.2) | 0.09 | (0.03-0.2) | 1.000 (p<0.001) | 1.000 (p<0.001) |
| MBC IgG2 CD20+CD21+CD27- | 0.003 | (0.0006-0.005) | 0.001 | (0.000-0.002) | 0.004 | (0.0006-0.01) | 0.623 (p=0.112) | 0.691 (p=0.081) |
| MBC IgG2 CD20++CD21- | 0.004 | (0.002-0.006) | 0.0002 | (0.000-0.0008) | 0.004 | (0.001-0.007) | 0.814 (p=0.036) | 0.64 (p=0.104) |
| MBC IgG2 CD20++CD21-CD24+ | 0.002 | (0.001-0.004) | 0.0002 | (0.000-0.0008) | 0.002 | (0.000-0.006) | 0.974 (p=0.002) | 0.533 (p=0.162) |
| MBC IgG2 CD20++CD21-CD24+CD27+ | 0.002 | (0.001-0.003) | ND |  | 0.001 | (0.000-0.006) | ND | 0.874 (p=0.065) |
| MBC IgG2 CD20++CD21-CD24+CD27- | 0.0005 | (0.000-0.001) | 0.0002 | (0.000-0.0008) | 0.0004 | (0.000-0.002) | 0.880 (p=0.018) | 0.916 (p=0.043) |
| MBC IgG2 CD20++CD21-CD24- | 0.002 | (0.0008-0.003) | ND |  | 0.002 | (0.001-0.004) | ND | 0.960 (p=0.003) |
| MBC IgG2 CD20++CD21-CD24-CD27+ | 0.0006 | (0.000-0.001) | ND |  | 0.0003 | (0.000-0.001) | ND | 0.582 (p=0.448) |
| MBC IgG2 CD20++CD21-CD24-CD27- | 0.001 | (0.000-0.002) | ND |  | 0.002 | (0.0006-0.004) | ND | 0.951 (p=0.025) |
| MBC IgG3+ | 0.05 | (0.02-0.09) | 0.05 | (0.01-0.1) | 0.05 | (0.02-0.1) | 0.986 (p=0.001) | 0.990 (p<0.001) |
| MBC IgG3 CD20+CD21+ | 0.03 | (0.01-0.06) | 0.04 | (0.01-0.06) | 0.03 | (0.009-0.07) | 0.962 (p=0.003) | 0.990 (p<0.001) |
| MBC IgG3 CD20+CD21+CD27+ | 0.02 | (0.006-0.05) | 0.03 | (0.009-0.06) | 0.02 | (0.007-0.05) | 0.964 (p=0.003) | 0.992 (p<0.001) |
| MBC IgG3 CD20+CD21+CD27- | 0.01 | (0.004-0.02) | 0.006 | (0.002-0.008) | 0.01 | (0.002-0.02) | 0.764 (p=0.053) | 0.903 (p=0.013) |
| MBC IgG3 CD20++CD21- | 0.01 | (0.005-0.03) | 0.01 | (0.003-0.03) | 0.01 | (0.007-0.03) | 0.996 (p<0.001) | 0.982 (p=0.001) |
| MBC IgG3 CD20++CD21-CD24+ | 0.006 | (0.003-0.01) | 0.005 | (0.000-0.01) | 0.006 | (0.003-0.01) | 0.924 (p=0.009) | 0.970 (p=0.002) |
| MBC IgG3 CD20++CD21-CD24+CD27+ | 0.003 | (0.002-0.006) | 0.001 | (0.000-0.005) | 0.003 | (0.002-0.005) | 0.931 (p=0.008) | 0.953 (p=0.004) |
| MBC IgG3 CD20++CD21-CD24+CD27- | 0.003 | (0.002-0.006) | 0.003 | (0.000-0.01) | 0.003 | (0.002-0.006) | 0.897 (p=0.015) | 0.922 (p=0.010) |
| MBC IgG3 CD20++CD21-CD24- | 0.007 | (0.002-0.02) | 0.007 | (0.0009-0.02) | 0.008 | (0.003-0.02) | 0.951 (p=0.005) | 0.988 (p=0.001) |
| MBC IgG3 CD20++CD21-CD24-CD27+ | 0.001 | (0.0004-0.004) | 0.000 | (0.000-0.0002) | 0.001 | (0.000-0.003) | 0.906 (p=0.012) | 0.976 (p=0.012) |
| MBC IgG3 CD20++CD21-CD24-CD27- | 0.005 | (0.002-0.01) | 0.007 | (0.0009-0.02) | 0.006 | (0.002-0.02) | 0.964 (p=0.003) | 0.980 (p=0.001) |
| MBC IgG4+ | 0.01 | (0.004-0.03) | 0.01 | (0.002-0.03) | 0.01 | (0.004-0.03) | 0.976 (p=0.002) | 0.984 (p=0.001) |
| MBC IgG4 CD20+CD21+ | 0.01 | (0.004-0.02) | 0.01 | (0.002-0.03) | 0.01 | (0.004-0.03) | 0.978 (p=0,001) | 0.994 (p<0.001) |
| MBC IgG4 CD20+CD21+CD27+ | 0.01 | (0.004-0.02) | 0.01 | (0.002-0.03) | 0.01 | (0.004-0.03) | 0.998 (p<0.001) | 0.998 (p<0.001) |
| MBC IgG4 CD20+CD21+CD27- | 0.001 | (0.000-0.004) | ND |  | ND |  | ND | ND |
| MBC IgG4 CD20++CD21- | 0.0009 | (0.000-0.002) | 0.0003 | (0.000-0.001) | 0.0004 | (0.000-0.001) | 0.769 (p=0.051) | 0.020 (p=0.982) |
| MBC IgG4 CD20++CD21-CD24+ | 0.0009 | (0.000-0.002) | 0.0003 | (0.000-0.001) | 0.0004 | (0.000-0.001) | 0.513 (p=0.174) | 0.020 (p=0.982) |
| MBC IgG4 CD20++CD21-CD24+CD27+ | 0.0007 | (0.000-0.001) | 0.0003 | (0.000-0.001) | 0.0003 | (0.000-0.001) | 0.542 (p=0.156) | 0.081 (p=0.643) |
| MBC IgG4 CD20++CD21-CD24+CD27- | 0.0002 | (0.000-0.0005) | ND |  | 0.0001 | (0.000-0.0006) | ND | 0.163 (p=0.596) |
| MBC IgA1+ | 0.2 | (0.04-0.3) | 0.2 | (0.04-0.3) | 0.2 | (0.04-0.3) | 0.998 (p<0.001) | 0.998 (p<0.001) |
| MBC IgA1 CD20+CD21+ | 0.2 | (0.04-0.3) | 0.2 | (0.04-0.3) | 0.2 | (0.04-0.3) | 0.998 (p<0.001) | 0.998 (p<0.001) |
| MBC IgA1 CD20+CD21+CD27+ | 0.1 | (0.03-0.3) | 0.2 | (0.04-0.3) | 0.2 | (0.04-0.3) | 0.994 (p<0.001) | 1.000 (p<0.001) |
| MBC IgA1 CD20+CD21+CD27- | 0.01 | (0.003-0.02) | 0.004 | (0.000-0.008) | 0.02 | (0.004-0.04) | 0.701 (p=0.077) | 0.848 (p=0.026) |
| MBC IgA1 CD20++CD21- | 0.01 | (0.003-0.02) | 0.009 | (0.002-0.02) | 0.01 | (0.003-0.02) | 0.992 (p<0.001) | 0.972 (p=0.002) |
| MBC IgA1 CD20++CD21- CD24+ | 0.003 | (0.0009-0.008) | ND |  | 0.004 | (0.0007-0.01) | ND | 0.897 (p=0.015) |
| MBC IgA1 CD20++CD21- CD24+CD27+ | 0.002 | (0.0005-0.006) | ND |  | 0.003 | (0.000-0.005) | ND | 0.618 (p=0.115) |
| MBC IgA1 CD20++CD21- CD24+CD27- | 0.001 | (0.0004-0.002) | ND |  | 0.002 | (0.0005-0.005) | ND | 0.958 (p=0.004) |
| MBC IgA1 CD20++CD21- CD24- | 0.007 | (0.002-0.02) | 0.009 | (0.002-0.02) | 0.007 | (0.002-0.01) | 0.992 (p<0.001) | 0.988 (p=0.001) |
| MBC IgA1 CD20++CD21- CD24-CD27+ | 0.002 | (0.0005-0.003) | 0.0009 | (0.0004-0.002) | 0.002 | (0.0006-0.003) | 0.153 (p=0.515) | 0.988 (p=0.001) |
| MBC IgA1 CD20++CD21- CD24-CD27- | 0.006 | (0.001-0.01) | 0.008 | (0.001-0.02) | 0.006 | (0.002-0.01) | 0.978 (p=0.001) | 0.988 (p=0.001) |
| MBC IgA2+ | 0.06 | (0.01-0.1) | 0.07 | (0.01-0.1) | 0.07 | (0.01-0.1) | 0.998 (p<0.001) | 0.998 (p<0.001) |
| MBC IgA2 CD20+CD21+ | 0.06 | (0.01-0.1) | 0.07 | (0.01-0.1) | 0.07 | (0.01-0.1) | 0.998 (p<0.001) | 0.998 (p<0.001) |
| MBC IgA2 CD20+CD21+CD27+ | 0.06 | (0.009-0.1) | 0.07 | (0.01-0.1) | 0.07 | (0.01-0.1) | 0.992 (p<0.001) | 0.998 (p<0.001) |
| MBC IgA2 CD20+CD21+CD27- | 0.003 | (0.0008-0.009) | 0.0009 | (0.000-0.003) | 0.002 | (0.000-0.009) | 0.624 (p=0.112) | 0.834 (p=0.087) |
| MBC IgA2 CD20++CD21- | 0.001 | (0.000-0.003) | ND |  | 0.0006 | (0.000-0.002) | ND | 0.726 (p=0.148) |
| MBC IgA2 CD20++CD21-CD24+ | 0.0005 | (0.000-0.001) | ND |  | ND |  | ND | ND |
| MBC IgA2 CD20++CD21-CD24+CD27+ | 0.0003 | (0.000-0.001) | ND |  | ND |  | ND | ND |
| MBC IgA2 CD20++CD21-CD24+CD27- | ND |  | ND |  | ND |  | ND | ND |
| MBC IgA2 CD20++CD21-CD24- | 0.0005 | (0.000-0.001) | ND |  | 0.0006 | (0.000-0.002) | ND | 0.766 (p=0.125) |
| MBC IgA2 CD20++CD21-CD24-CD27+ | 0.0001 | (0.000-0.0005) | ND |  | 0.0002 | (0.000-0.0009) | ND | 0.063 (p=0.685) |
| MBC IgA2 CD20++CD21-CD24-CD27- | 0.0002 | (0.000-0.0008) | ND |  | 0.0005 | (0.000-0.001) | ND | 0.835 (p=0.086) |
| MBC IgD+ | 0.006 | (0.000-0.02) | 0.002 | (0.000-0.009) | 0.004 | (0.000-0.01) | 0.947 (p=0.005) | 0.925 (p=0.009) |
| MBC IgD CD20+CD21+ | 0.004 | (0.000-0.01) | 0.002 | (0.000-0.007) | 0.003 | (0.000-0.008) | 0.927 (p=0.009) | 0.908 (p=0.012) |
| MBC IgD CD20++CD21- | 0.003 | (0.000-0.009) | 0.0004 | (0.000-0.002) | 0.0004 | (0.000-0.002) | 0.972 (p=0.002) | 0.953 (p=0.005) |
| MBC IgD CD20++CD21-CD24+ | 0.0005 | (0.000-0.001) | ND |  | ND |  | ND | ND |
| MBC IgD CD20++CD21-CD24- | 0.002 | (0.000-0.008) | 0.0004 | (0.000-0.002) | 0.0004 | (0.000-0.002) | 0.949 (p=0.005) | 0.955 (p=0.004) |
| MBC IgH- | 0.02 | (0.004-0.03) | 0.01 | (0.003-0.02) | 0.02 | (0.005-0.03) | 0.832 (p=0.031) | 0.001 (p=0.974) |
| Plasma Cells | 0.03 | (0.008-0.06) | 0.04 | (0.009-0.07) | 0.04 | (0.01-0.07) | 1.000 (p<0.001) | 0.968 (p=0.002) |
| PC IgM+ | 0.006 | (0.0009-0.009) | 0.006 | (0.0002-0.009) | 0.006 | (0.001-0.01) | 0.941 (p=0.006) | 0.972 (p=0.002) |
| PC IgM CD20+CD138 | 0.001 | (0.0004-0.002) | 0.0002 | (0.000-0.0007) | 0.001 | (0.000-0.002) | ND | 0.684 (p=0.084) |
| PC IgM CD20-CD138- | 0.003 | (0.0006-0.005) | 0.003 | (0.000-0.008) | 0.003 | (0.0005-0.005) | 0.857 (p=0.024) | 0.859 (p=0.024) |
| PC IgM CD20-CD138+ | 0.001 | (0.000-0.003) | 0.002 | (0.000-0.007) | 0.002 | (0.000-0.006) | 0.819 (p=0.035) | 0.968 (p=0.016) |
| PC IgG1+ | 0.004 | (0.002-0.008) | 0.004 | (0.0003-0.009) | 0.005 | (0.0008-0.01) | 0.914 (p=0.011) | 0.941 (p=0.007) |
| PC IgG1 CD20+CD138- | 0.0006 | (0.000-0.001) | 0.0001 | (0.000-0.0007) | 0.0007 | (0.000-0.002) | ND | 0.235 (p=0.515) |
| PC IgG1 CD20-CD138- | 0.003 | (0.001-0.006) | 0.003 | (0.0003-0.007) | 0.003 | (0.0008-0.006) | 0.916 (p=0.010) | 0.955 (p=0.004) |
| PC IgG1 CD20-CD138+ | 0.0005 | (0.000-0.001) | 0.0008 | (0.000-0.002) | 0.001 | (0.000-0.003) | 0.465 (p=0.204) | 0.801 (p=0.105) |
| PC IgG2+ | 0.004 | (0.001-0.008) | 0.004 | (0.001-0.008) | 0.004 | (0.002-0.008) | 0.978 (p=0.001) | 0.980 (p=0.001) |
| PC IgG2 CD20+CD138- | 0.0005 | (0.000-0.001) | 0.0008 | (0.000-0.003) | 0.0007 | (0.000-0.001) | 0.666 (p=0.092) | 0.904 (p=0.049) |
| PC IgG2 CD20-CD138- | 0.003 | (0.001-0.006) | 0.003 | (0.0005-0.006) | 0.002 | (0.0008-0.005) | 0.935 (p=0.007) | 0.970 (p=0.002) |
| PC IgG2 CD20-CD138+ | 0.0004 | (0.000-0.0008) | 0.0008 | (0.000-0.001) | 0.001 | (0.0008-0.002) | 0.002 (p=0.947) | 0.054 (p=0.767) |
| PC IgG3+ | ND |  | ND |  | ND |  | ND | ND |
| PC IgG3 CD20+CD138- | ND |  | ND |  | ND |  | ND | ND |
| PC IgG3 CD20-CD138- | ND |  | ND |  | ND |  | ND | ND |
| PC IgG3 CD20-CD138+ | ND |  | ND |  | ND |  | ND | ND |
| PC IgG4+ | 0.0006 | (0.000-0.0011) | ND |  | 0.0004 | (0.000-0.001) | ND | 0.618 (p=0.115) |
| PC IgG4 CD20+CD138- | ND |  | ND |  | ND |  | ND | ND |
| PC IgG4 CD20-CD138- | 0.0005 | (0.000-0.0008) | ND |  | 0.0004 | (0.000-0.0008) | ND | 0.029 (p=0.830) |
| PC IgG4 CD20-CD138+ | 0.0001 | (0.000-0.0003) | ND |  | 0.0001 | (0.000-0.0004) | ND | 1.000 (p<0.001) |
| PC IgA1+ | 0.01 | (0.002-0.02) | 0.02 | (0.006-0.03) | 0.01 | (0.004-0.02) | 0.970 (p=0.002) | 0.958 (p=0.004) |
| PC IgA1 CD20+CD138- | 0.001 | (0.000-0.002) | 0.002 | (0.000-0.004) | 0.003 | (0.001-0.006) | 0.521 (p=0.169) | 0.876 (p=0.064) |
| PC IgA1 CD20-CD138- | 0.008 | (0.002-0.01) | 0.02 | (0.006-0.03) | 0.006 | (0.002-0.01) | 0.964 (p=0.003) | 0.901 (p=0.014) |
| PC IgA1 CD20-CD138+ | 0.001 | (0.0003-0.002) | 0.0005 | (0.000-0.003) | 0.005 | (0.0008-0.007) | 0.362 (p=0.283) | 0.325 (p=0.316) |
| PC IgA2+ | 0.006 | (0.0009-0.02) | 0.01 | (0.002-0.02) | 0.009 | (0.002-0.02) | 0.984 (p=0.001) | 0.910 (p=0.012) |
| PC IgA2 CD20+CD138- | 0.0004 | (0.000-0.001) | 0.002 | (0.000-0.006) | 0.002 | (0.0005-0.005) | 0.637 (p=0.105) | 0.593 (p=0.441) |
| PC IgA2 CD20-CD138- | 0.006 | (0.0009-0.02) | 0.008 | (0.002-0.02) | 0.005 | (0.001-0.01) | 0.828 (p=0.032) | 0.994 (p<0.001) |
| PC IgA2 CD20-CD138+ | 0.0003 | (0.000-0.001) | 0.0002 | (0.000-0.0007) | 0.001 | (0.000-0.004) | 0.019 (p=0.825) | 0.994 (p<0.001) |
| PC IgD+ | 0.0001 | (0.000-0.0006) | 0.0002 | (0.000-0.001) | 0.0002 | (0.000-0.001) | 0.937 (p=0.007) | 1.000 (p<0.001) |
| PC IgD CD20+CD138- | ND |  | ND |  | ND |  | ND | ND |
| PC IgD CD20-CD138- | 0.0001 | (0.000-0.0006) | 0.0002 | (0.000-0.001) | 0.0002 | (0.000-0.0009) | 0.937 (p=0.007) | 1.000 (p<0.001) |
| PC IgD CD20-CD138+ | ND |  | ND |  | ND |  | ND | ND |

Results expressed as median percent (range) values and as coefficient of determination R² (p-values). Five files reviewed by the same expert were used to compare the two AG&I algorithms. Coefficient of determination (R²) and p values were calculated using SPSS (v.18.0.0; SPSS-IBM, Armonk, NY). Values marked in green and in blue represent a significantly better correlation for the supervised algorithm (Algorithm 2) vs unsupervised algorithm (Algorithm 1) respectively.

**Supplementary Table 3-** Reproducibility of the conventional manual gating approach vs the automated gating and identification approach for the identification of cell populations in normal blood samples from the validation (n=147) set of FCS datafile stained with the EuroFlow BIgH-IMM antibody combination.

| **Cell populations** | Manual analysis (%CV) | AG&I (%CV) | Coefficient of determination (R²) | Mean normalized bias |
| --- | --- | --- | --- | --- |
| Nucleated cells | 100 | 100 | 0.967 (p<0.001) | 5 |
| Myeloid cells | 67 (0.033-92) | 64 (31-92) | 0.962 (p<0.001) | 7 |
| Eosinophils | 2.2 (<0.001-22) | 2.2 (0.1-21) | 0.989 (p<0.001) | -1 |
| Neutrophils | 57 (0.014-87) | 54 (14-86) | 0.956 (p<0.001) | 8 |
| Monocyte | 6.6 (0.004-16) | 6.8 (3.3-15) | 0.977 (p<0.001) | 6 |
| Lymphocytes | 32 (7.8-63) | 35 (8.4-66) | 0.994 (p<0.001) | -1 |
| T/NK lymphocytes | 27 (7.3-60) | 29 (7.9-63) | 0.993 (p<0.001) | -1 |
| B-lymphocytes | 4.5 (0.5-12) | 4.9 (0.5-13) | 0.997 (p<0.001) | -1 |
| Pre-GC B cells | 3.4 (0.2-9.4) | 3.6 (0.3-9.8) | 0.993 (p<0.001) | 1 |
| Immature B cells | 0.2 (<0.001-2.7) | 0.2 (<0.001.-3.2) | 0.883 (p<0.001) | 2 |
| Naive CD5+ B cells | 0.5 (0.01-3.8) | 0.7 (<0.001-3.7) | 0.712 (p<0.001) | -26 |
| Naïve B cells | 2.4 (0.2-8.5) | 2.2 (0.3-7.3) | 0.923 (p<0.001) | 12 |
| Naive B CD20+CD21+ | 2.4 (0.1-8.5) | 2.2 (0.3-7.3) | 0.921 (p<0.001) | 12 |
| Naive B CD20+CD21+CD62L+ | 0.9 (<0.001-6.2) | 0.7 (<0.001-4) | 0.893 (p<0.001) | 27 |
| Naive B CD20+CD21+CD62L- | 0.9 (<0.001-5.6) | 1 (<0.001-4.9) | 0.904 (p<0.001) | 19 |
| Naive B CD20++CD21- | 0.04 (<0.001-0.2) | 0.04 (<0.001-0.5) | 0.917 (p<0.001) | -2 |
| Memory B-Cells (MBC) | 1 (<0.001-4.2) | 1.2 (<0.001-4.7) | 0.986 (p<0.001) | -3 |
| MBC IgMD+ | 0.5 (<0.001-1.6) | 0.6 (<0.001-2.1) | 0.931 (p<0.001) | -8 |
| MBC IgMD CD20+CD21+ | 0.4 (<0.001-1.5) | 0.5 (<0.001-1.9) | 0.925 (p<0.001) | -9 |
| MBC IgMD CD20++CD21- | 0.03 (<0.001-0.3) | 0.03 (<0.001-0.4) | 0.886 (p<0.001) | 10 |
| MBC IgMD CD20++CD21-CD24+ | 0.03 (<0.001-0.3) | 0.03 (<0.001-0.4) | 0.844 (p<0.001) | 9 |
| MBC IgMD CD20++CD21-CD24- | 0.004 (<0.001-0.1) | 0.004 (<0.001-0.08) | 0.851 (p<0.001) | 39 |
| MBC IgG1+ | 0.2 (<0.001-1.5) | 0.2 (<0.001-1.5) | 0.996 (p<0.001) | 0 |
| MBC IgG1 CD20+CD21+ | 0.2 (<0.001-1.3) | 0.2 (<0.001-1.3) | 0.996 (p<0.001) | -2 |
| MBC IgG1 CD20+CD21+CD27+ | 0.1 (<0.001-1.1) | 0.1 (<0.001-1.1) | 0.964 (p<0.001) | 8 |
| MBC IgG1 CD20+CD21+CD27- | 0.04 (<0.001-0.5) | 0.05 (<0.001-0.6) | 0.837 (p<0.001) | -11 |
| MBC IgG1 CD20++CD21- | 0.03 (<0.001-0.2) | 0.03 (<0.001-0.2) | 0.979 (p<0.001) | -6 |
| MBC IgG1 CD20++CD21-CD24+ | 0.01 (<0.001-0.1) | 0.01 (<0.001-0.1) | 0.918 (p<0.001) | -13 |
| MBC IgG1 CD20++CD21-CD24+CD27+ | 0.007 (<0.001-0.06) | 0.009 (<0.001-0.08) | 0.892 (p<0.001) | -14 |
| MBC IgG1 CD20++CD21-CD24+CD27- | 0.004 (<0.001-0.06) | 0.004 (<0.001-0.06) | 0.887 (p<0.001) | 1 |
| MBC IgG1 CD20++CD21-CD24- | 0.01 (<0.001-0.1) | 0.02 (<0.001-0.1) | 0.973 (p<0.001) | -3 |
| MBC IgG1 CD20++CD21-CD24-CD27+ | 0.004 (<0.001-0.04) | 0.003 (<0.001-0.05) | 0.886 (p<0.001) | 7 |
| MBC IgG1 CD20++CD21-CD24-CD27- | 0.01 (<0.001-0.1) | 0.01 (<0.001-0.1) | 0.982 (p<0.001) | -4 |
| MBC IgG2+ | 0.04 (<0.001-0.2) | 0.04 (<0.001-0.3) | 0.994 (p<0.001) | -3 |
| MBC IgG2 CD20+CD21+ | 0.04 (<0.001-0.2) | 0.04 (<0.001-0.3) | 0.994 (p<0.001) | -5 |
| MBC IgG2 CD20+CD21+CD27+ | 0.04 (<0.001-0.2) | 0.04 (<0.001-0.3) | 0.991 (p<0.001) | -4 |
| MBC IgG2 CD20+CD21+CD27- | 0.002 (<0.001-0.03) | <0.001 (<0.001-0.02) | 0.823 (p<0.001) | 21 |
| MBC IgG2 CD20++CD21- | 0.002 (<0.001-0.02) | 0.001 (<0.001-0.03) | 0.854 (p<0.001) | 48 |
| MBC IgG2 CD20++CD21-CD24+ | <0.001 (<0.001-0.02) | <0.001 (<0.001-0.02) | 0.712 (p<0.001) | 35 |
| MBC IgG2 CD20++CD21-CD24+CD27+ | <0.001 (<0.001-0.02) | <0.001 (<0.001-0.02) | 0.823 (p<0.001) | 25 |
| MBC IgG2 CD20++CD21-CD24+CD27- | <0.001 (<0.001-0.006) | <0.001 (<0.001-0.005) | 0.642 (p<0.001) | 3 |
| MBC IgG2 CD20++CD21-CD24- | <0.001 (<0.001-0.01) | <0.001 (<0.001-0.02) | 0.832 (p<0.001) | 19 |
| MBC IgG2 CD20++CD21-CD24-CD27+ | <0.001 (<0.001-0.003) | <0.001 (<0.001-0.004) | 0.48 (p<0.001) | 4 |
| MBC IgG2 CD20++CD21-CD24-CD27- | <0.001 (<0.001-0.01) | <0.001 (<0.001-0.01) | 0.887 (p<0.001) | 6 |
| MBC IgG3+ | 0.04 (<0.001-0.2) | 0.04 (<0.001-0.2) | 0.987 (p<0.001) | 5 |
| MBC IgG3 CD20+CD21+ | 0.03 (<0.001-0.2) | 0.03 (<0.001-0.2) | 0.988 (p<0.001) | 4 |
| MBC IgG3 CD20+CD21+CD27+ | 0.02 (<0.001-0.1) | 0.02 (<0.001-0.1) | 0.958 (p<0.001) | 1 |
| MBC IgG3 CD20+CD21+CD27- | 0.01 (<0.001-0.1) | 0.008 (<0.001-0.1) | 0.863 (p<0.001) | 16 |
| MBC IgG3 CD20++CD21- | 0.008 (<0.001-0.08) | 0.009 (<0.001-0.09) | 0.960 (p<0.001) | -2 |
| MBC IgG3 CD20++CD21-CD24+ | 0.003 (<0.001-0.03) | 0.003 (<0.001-0.03) | 0.862 (p<0.001) | 17 |
| MBC IgG3 CD20++CD21-CD24+CD27+ | 0.002 (<0.001-0.02) | 0.001 (<0.001-0.02) | 0.864 (p<0.001) | 19 |
| MBC IgG3 CD20++CD21-CD24+CD27- | 0.001 (<0.001-0.02) | <0.001 (<0.001-0.03) | 0.751 (p<0.001) | 16 |
| MBC IgG3 CD20++CD21-CD24- | 0.004 (<0.001-0.05) | 0.005 (<0.001-0.06) | 0.954 (p<0.001) | -10 |
| MBC IgG3 CD20++CD21-CD24-CD27+ | <0.001 (<0.001-0.02) | <0.001 (<0.001-0.02) | 0.901 (p<0.001) | -1 |
| MBC IgG3 CD20++CD21-CD24-CD27- | 0.003 (<0.001-0.03) | 0.004 (<0.001-0.04) | 0.953 (p<0.001) | -12 |
| MBC IgG4+ | 0.004 (<0.001-0.05) | 0.004 (<0.001-0.05) | 0.964 (p<0.001) | 17 |
| MBC IgG4 CD20+CD21+ | 0.004 (<0.001-0.05) | 0.004 (<0.001-0.05) | 0.965 (p<0.001) | 15 |
| MBC IgG4 CD20+CD21+CD27+ | 0.003 (<0.001-0.03) | 0.004 (<0.001-0.04) | 0.829 (p<0.001) | 1 |
| MBC IgG4 CD20+CD21+CD27- | <0.001 (<0.001-0.03) | <0.001 (<0.001-0.009) | 0.543 (p<0.001) | 18 |
| MBC IgG4 CD20++CD21- | <0.001 (<0.001-0.005) | <0.001 (<0.001-0.02) | 0.706 (p<0.001) | 6 |
| MBC IgG4 CD20++CD21-CD24+ | <0.001 (<0.001-0.005) | <0.001 (<0.001-0.006) | 0.757 (p<0.001) | 5 |
| MBC IgG4 CD20++CD21-CD24+CD27+ | <0.001 (<0.001-0.003) | <0.001 (<0.001-0.006) | 0.849 (p<0.001) | -2 |
| MBC IgG4 CD20++CD21-CD24+CD27- | <0.001 (<0.001-0.004) | <0.001 (<0.001-0.004) | 1.000 (p<0.001) | 0 |
| MB IgA1+ | 0.1 (<0.001-0.6) | 0.1 (<0.001-0.6) | 0.996 (p<0.001) | -5 |
| MBC IgA1 CD20+CD21+ | 0.1 (<0.001-0.5) | 0.1 (<0.001-0.5) | 0.994 (p<0.001) | -8 |
| MBC IgA1 CD20+CD21+CD27+ | 0.1 (<0.001-0.5) | 0.1 (<0.001-0.5) | 0.991 (p<0.001) | -1 |
| MBC IgA1 CD20+CD21+CD27- | 0.01 (<0.001-0.07) | 0.02 (<0.001-0.1) | 0.726 (p<0.001) | -31 |
| MBC IgA1 CD20++CD21- | 0.008 (<0.001-0.1) | 0.01 (<0.001-0.1) | 0.963 (p<0.001) | -9 |
| MBC IgA1 CD20++CD21- CD24+ | 0.003 (<0.001-0.05) | 0.003 (<0.001-0.06) | 0.888 (p<0.001) | 3 |
| MBC IgA1 CD20++CD21-CD24+CD27+ | 0.002 (<0.001-0.04) | 0.002 (<0.001-0.06) | 0.883 (p<0.001) | -3 |
| MBC IgA1 CD20++CD21-CD24+CD27- | <0.001 (<0.001-0.007) | <0.001 (<0.001-0.006) | 0.813 (p<0.001) | 15 |
| MBC IgA1 CD20++CD21- CD24- | 0.005 (<0.001-0.06) | 0.007 (<0.001-0.06) | 0.956 (p<0.001) | -9 |
| MBC IgA1 CD20++CD21-CD24-CD27+ | 0.001 (<0.001-0.01) | <0.001 (<0.001-0.01) | 0.810 (p<0.001) | 0 |
| MBC IgA1 CD20++CD21- CD24-CD27- | 0.003 (<0.001-0.05) | 0.004 (<0.001-0.05) | 0.964 (p<0.001) | -10 |
| MBC IgA2+ | 0.04 (<0.001-0.2) | 0.04 (<0.001-0.3) | 0.976 (p<0.001) | -2 |
| MBC IgA2 CD20+CD21+ | 0.03 (<0.001-0.2) | 0.04 (<0.001-0.3) | 0.973 (p<0.001) | -4 |
| MBC IgA2 CD20+CD21+CD27+ | 0.03 (<0.001-0.2) | 0.04 (<0.001-0.2) | 0.969 (p<0.001) | -6 |
| MBC IgA2 CD20+CD21+CD27- | 0.003 (<0.001-0.04) | <0.001 (<0.001-0.03) | 0.562 (p<0.001) | 44 |
| MBC IgA2 CD20++CD21- | <0.001 (<0.001-0.007) | <0.001 (<0.001-0.01) | 0.368 (p<0.001) | 48 |
| MBC IgA2 CD20++CD21-CD24+ | <0.001 (<0.001-0.005) | <0.001 (<0.001-0.01) | 0.362 (p<0.001) | 15 |
| MBC IgA2 CD20++CD21-CD24+CD27+ | <0.001 (<0.001-0.005) | <0.001 (<0.001-0.01) | 0.393 (p<0.001) | 12 |
| MBC IgA2 CD20++CD21-CD24+CD27- | - | - | - | - |
| MBC IgA2 CD20++CD21-CD24- | <0.001 (<0.001-0.005) | <0.001 (<0.001-0.006) | 0.044 (p<0.001) | 16 |
| MBC IgA2 CD20++CD21-CD24-CD27+ | <0.001 (<0.001-0.003) | <0.001 (<0.001-0.006) | 0.123 (p<0.001) | -1 |
| MBC IgA2 CD20++CD21-CD24-CD27- | <0.001 (<0.001-0.002) | <0.001 (<0.001-0.004) | 0.052 (p<0.001) | 5 |
| MBC IgD+ | 0.003 (<0.001-0.3) | 0.003 (<0.001-0.3) | 0.990 (p<0.001) | 8 |
| MBC IgD CD20+CD21+ | 0.003 (<0.001-0.3) | 0.003 (<0.001-0.3) | 0.991 (p<0.001) | -7 |
| MBC IgD CD20++CD21- | <0.001 (<0.001-0.01) | <0.001 (<0.001-0.02) | 0.507 (p<0.001) | 41 |
| MBC IgD CD20++CD21-CD24+ | <0.001 (<0.001-0.007) | <0.001 (<0.001-0.01) | 0.619 (p<0.001) | -4 |
| MBC IgD CD20++CD21-CD24- | <0.001 (<0.001-0.01) | <0.001 (<0.001-0.006) | 0.264 (p<0.001) | 27 |
| MBC IgH- | 0.01 (<0.001-0.08) | 0.02 (<0.001-0.2) | 0.643 (p<0.001) | -50 |
| Plasma Cells | 0.05 (<0.001-0.8) | 0.06 (<0.001-0.8) | 0.996 (p<0.001) | 0 |
| PC IgM+ | 0.005 (<0.001-0.1) | 0.005 (<0.001-0.1) | 0.955 (p<0.001) | 8 |
| PC CD20+ CD138- IgM+ | <0.001 (<0.001-0.003) | <0.001 (<0.001-0.004) | 0.610 (p<0.001) | -4 |
| PC CD20- CD138- IgM+ | 0.004 (<0.001-0.05) | 0.003 (<0.001-0.05) | 0.967 (p<0.001) | 1 |
| PC CD20- CD138+ IgM+ | 0.001 (<0.001-0.08) | 0.002 (<0.001-0.07) | 0.950 (p<0.001) | 2 |
| PC IgG1+ | 0.006 (<0.001-0.4) | 0.006 (<0.001-0.4) | 0.977 (p<0.001) | 4 |
| PC CD20+ CD138- IgG1+ | <0.001 (<0.001-0.009) | <0.001 (<0.001-0.003) | 0.769 (p<0.001) | 8 |
| PC CD20- CD138- IgG1+ | 0.004 (<0.001-0.2) | 0.004 (<0.001-0.2) | 0.965 (p<0.001) | 0 |
| PC CD20- CD138+ IgG1+ | 0.001 (<0.001-0.2) | 0.001 (<0.001-0.2) | 0.968 (p<0.001) | -6 |
| PC IgG2+ | 0.003 (<0.001-0.07) | 0.003 (<0.001-0.07)) | 0.970 (p<0.001) | 8 |
| PC CD20+ CD138- IgG2+ | <0.001 (<0.001-0.002) | <0.001 (<0.001-0.002) | 0.429 (p<0.001) | 0 |
| PC CD20- CD138- IgG2+ | 0.003 (<0.001-0.05) | 0.003 (<0.001-0.04) | 0.954 (p<0.001) | 5 |
| PC CD20- CD138+ IgG2+ | <0.001 (<0.001-0.03) | <0.001 (<0.001-0.03) | 0.954 (p<0.001) | -4 |
| PC IgG3+ | <0.001 (<0.001-0.1) | <0.001 (<0.001-0.1) | 0.933 (p<0.001) | 11 |
| PC CD20+ CD138- IgG3+ | - | - | - | - |
| PC CD20- CD138- IgG3+ | <0.001 (<0.001-0.04) | <0.001 (<0.001-0.04) | 0.954 (p<0.001) | 6 |
| PC CD20- CD138+ IgG3+ | <0.001 (<0.001-0.06) | <0.001 (<0.001-0.06) | 0.976 (p<0.001) | 2 |
| PC IgG4+ | <0.001 (<0.001-0.02) | <0.001 (<0.001-0.02) | 0.976 (p<0.001) | 4 |
| PC CD20+ CD138- IgG4+ | - | - | - | - |
| PC CD20- CD138- IgG4+ | <0.001 (<0.001-0.01) | <0.001 (<0.001-0.009) | 0.935 (p<0.001) | -1 |
| PC CD20- CD138+ IgG4+ | <0.001 (<0.001-0.02) | <0.001 (<0.001-0.02) | 0.976 (p<0.001) | -2 |
| PC IgA1+ | 0.02 (<0.001-0.3) | 0.02 (<0.001-0.3) | 0.982 (p<0.001) | 1 |
| PC CD20+ CD138- IgA1+ | <0.001 (<0.001-0.01) | <0.001 (<0.001-0.01) | 0.650(p<0.001) | -16 |
| PC CD20- CD138- IgA1+ | 0.01 (<0.001-0.3) | 0.01 (<0.001-0.3) | 0.990 (p<0.001) | -2 |
| PC CD20- CD138+ IgA1+ | 0.002 (<0.001-0.1) | 0.003 (<0.001-0.1) | 0.894 (p<0.001) | -22 |
| PC IgA2+ | 0.005 (<0.001-0.07) | 0.006 (<0.001-0.08) | 0.952 (p<0.001) | 9 |
| PC CD20+ CD138- IgA2+ | <0.001 (<0.001-0.003) | <0.001 (<0.001-0.008) | 0.451 (p<0.001) | -24 |
| PC CD20- CD138- IgA2+ | 0.005 (<0.001-0.05) | 0.005 (<0.001-0.05) | 0.972 (p<0.001) | 1 |
| PC CD20- CD138+ IgA2+ | <0.001 (<0.001-0.02) | <0.001 (<0.001-0.04) | 0.641 (p<0.001) | -5 |
| PC IgD+ | <0.001 (<0.001-0.04) | <0.001 (<0.001-0.04) | 0.905 (p<0.001) | 5 |
| PC CD20+ CD138- IgD+ | <0.001 (<0.001-0.001) | <0.001 (<0.001-0.002) | 0.843 (p<0.001) | 1 |
| PC CD20- CD138- IgD+ | <0.001 (<0.001-0.04) | <0.001 (<0.001-0.04) | 0.996 (p<0.001) | -2 |
| PC CD20- CD138+ IgD+ | <0.001 (<0.001-0.01) | <0.001 (<0.001-0.008) | 0.948 (p<0.001) | 1 |

Results expressed as median frequency (range) among all nucleated cells. Comparison between expert-based manual analysis and the AG&I approach, high correlations (r^2^ ≥ 0.81; *p*-value ≤ 0.05), very good correlations (0.81 > r^2^ ≥ 0.64; *p*-value ≤ 0.05) and high degree of agreement (mean normalized bias (MNB) of ±20%) were obtained for 88% of the cell populations identified with the BIgH-IMM antibody combination. Coefficient of determination (R²) were calculated using SPSS (v.18.0.0; PSS-IBM, Armonk, NY) and MNB were calculated using Excel 2010 (Microsoft Corporation, Redmond, Washington, EE. UU). Values marked in red and in grey correspond to those correlations showing r^2^-values <0.64 and to r^2^-values ≥ 0.64 and <0.81, respectively. Results expressed as percentage of cells within all nucleated cells. **Abbreviations** (alphabetical order): AGI, automated gating and identification.
